# A scenario‐drafting study to explore potential future implementation pathways of circulating tumor DNA testing in oncology

**DOI:** 10.1002/1878-0261.13562

**Published:** 2024-01-11

**Authors:** Astrid Kramer, Carmen Rubio‐Alarcón, Daan van den Broek, Daan C. L. Vessies, Iris van't Erve, Gerrit A. Meijer, Geraldine R. Vink, Ed Schuuring, Remond J. A. Fijneman, Veerle M. H. Coupé, Valesca P. Retèl

**Affiliations:** ^1^ Department of Epidemiology and Data Science Amsterdam UMC The Netherlands; ^2^ Department of Pathology Netherlands Cancer Institute Amsterdam The Netherlands; ^3^ Department of Laboratory Medicine Netherlands Cancer Institute Amsterdam The Netherlands; ^4^ Department of Medical Oncology, University Medical Center Utrecht University of Utrecht The Netherlands; ^5^ Department of Research and Development IKNL Utrecht The Netherlands; ^6^ Department of Pathology and Medical Biology University Medical Center Groningen The Netherlands; ^7^ Department of Psychosocial Research and Epidemiology Netherlands Cancer Institute Amsterdam The Netherlands; ^8^ Erasmus School of Health Policy and Management Erasmus University Rotterdam The Netherlands

**Keywords:** clinical utility, ctDNA, HTA, implementation, oncology, scenarios

## Abstract

Circulating tumor DNA (ctDNA) detection has multiple promising applications in oncology, but the road toward implementation in clinical practice is unclear. We aimed to support the implementation process by exploring potential future pathways of ctDNA testing. To do so, we studied four ctDNA‐testing applications in two cancer types and elicited opinions from 30 ctDNA experts in the Netherlands. Our results showed that the current available evidence differed per application and cancer type. Tumor profiling and monitoring treatment response were found most likely to be implemented in non‐small cell lung cancer (NSCLC) within 5 years. For colorectal cancer, applications of ctDNA testing were found to be at an early stage in the implementation process. Demonstrating clinical utility was found a key aspect for successful implementation, but there was no consensus regarding the evidence requirements. The next step toward implementation is to define how clinical utility of biomarkers should be evaluated. Finally, these data indicate that specific challenges for each clinical application and tumor type should be appropriately addressed in a deliberative process involving all stakeholders to ensure implementation of ctDNA testing and timely access for patients.

## Graphical abstract

Analysis of circulating tumor DNA (ctDNA) is a promising technology with several applications in oncology. We identified the main challenges and opportunities to inform the implementation process in the Netherlands by eliciting opinions of experts. Next steps toward implementation of ctDNA is to define the evidence to prove clinical utility and to address the specific challenges for each application.

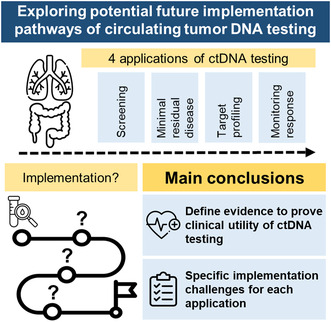

**Abbreviations**
cieBOD: Commissie Beoordeling Diagnostiek (Dutch advisory committee for diagnostics)
cieBOM: Commissie Beoordeling Oncologische Middelen (Dutch advisory committee for oncological agents)
COIN: ctDNA on the road to implementation in the Netherlands
CRC: colorectal cancer
ctDNA: circulating tumor DNA
DCCG: Dutch Colorectal Cancer Group
DORP: Dutch Oncology Research Platform
eHTA: early health technology assessment
HTA: health technology assessment
MRD: minimal residual disease
NSCLC: non‐small cell lung cancer
PLCRC: Prospective Dutch Colorectal Cancer Cohort
RCT: randomized clinical trial

## 1 Introduction

The use of biomarkers to detect and characterize cancer evolution over time is important to improve treatment decision‐making and intervene in the progression of disease [1, 2]. While microscopic evaluation of tumor tissue biopsies is the gold standard for cancer diagnostics, detection of cell‐free circulating tumor DNA (ctDNA) in liquid biopsies is a promising new technology that enables biomarker identification in a minimally invasive way [3]. CtDNA is composed of fragments of tumoral DNA present in blood or other body fluids, and it provides quantitative and qualitative information about a patient's tumor [4, 5]. This makes ctDNA testing useful in many different applications for a wide range of cancer types. For example, for identification of mutations to guide treatment decisions, real‐time monitoring of tumor evolution, minimal residual disease (MRD) detection, or even for screening purposes [6, 7, 8, 9, 10, 11, 12, 13]. The increasing interest in this technology is further reflected in the number of clinical studies currently including or investigating ctDNA testing, the establishment of numerous companies focused on ctDNA testing over the recent years, and the wide variety of ctDNA tests under development [14, 15]. The road for a promising technology such as ctDNA testing to reach implementation in clinical practice is long and complex [16]. Currently, the only ctDNA application included so far in clinical guidelines is the detection of resistance mechanisms in metastatic non‐small cell lung cancer (NSCLC), when tumor tissue is not available [17]. This application is currently used in routine diagnostics, albeit in a non‐coordinated way, leading to diversity in the analytical procedures amongst laboratories, and unequal access for patients to the latest diagnostic developments [18]. Therefore, elucidating the steps toward ctDNA implementation is an emerging need to bring ctDNA testing to patients in a structured way [19]. The Dutch multi‐disciplinary “CtDNA on the road to implementation in the Netherlands” (COIN)‐consortium (www.cfdna.nl/coin) is working to enable controlled, evidence‐based introduction of ctDNA testing in the Dutch healthcare system. An important task of COIN is to perform an early health technology assessment (eHTA) of ctDNA testing to comprehensively evaluate the expected impact of ctDNA testing in clinical practice [20]. HTA is often initiated in the last stages of implementation after the association between use of the test and change in health outcome (i.e. clinical utility) has been proven to inform policy and reimbursement decisions. Starting HTA earlier in the process (eHTA) helps to make evidence‐informed decisions to guide the development of the technology, design future clinical studies, and inform the implementation process [20, 21, 22]. As a result, the road toward implementation can be anticipated and steered toward timely access for patients.

However, the degree of uncertainty is higher when HTA is performed in an earlier stage, as fewer data are available [22]. Therefore, in this study, we aimed to explore potential future pathways to better understand the uncertainties, expectations, and potential barriers to plasma‐based ctDNA testing implementation. To do so, we elicited experts' opinions on developments in the field within 5 years in the Netherlands. We focused on two cancer types, NSCLC and colorectal cancer (CRC), and four different applications of ctDNA testing: early detection of cancer for screening, MRD detection, tumor profiling, and monitoring treatment evaluation. The results of this study can set the basis for future HTAs in the ctDNA field and can help elucidate the specific steps toward implementation needed in the near future.

## 2 Materials and methods

**Fig. 1:**
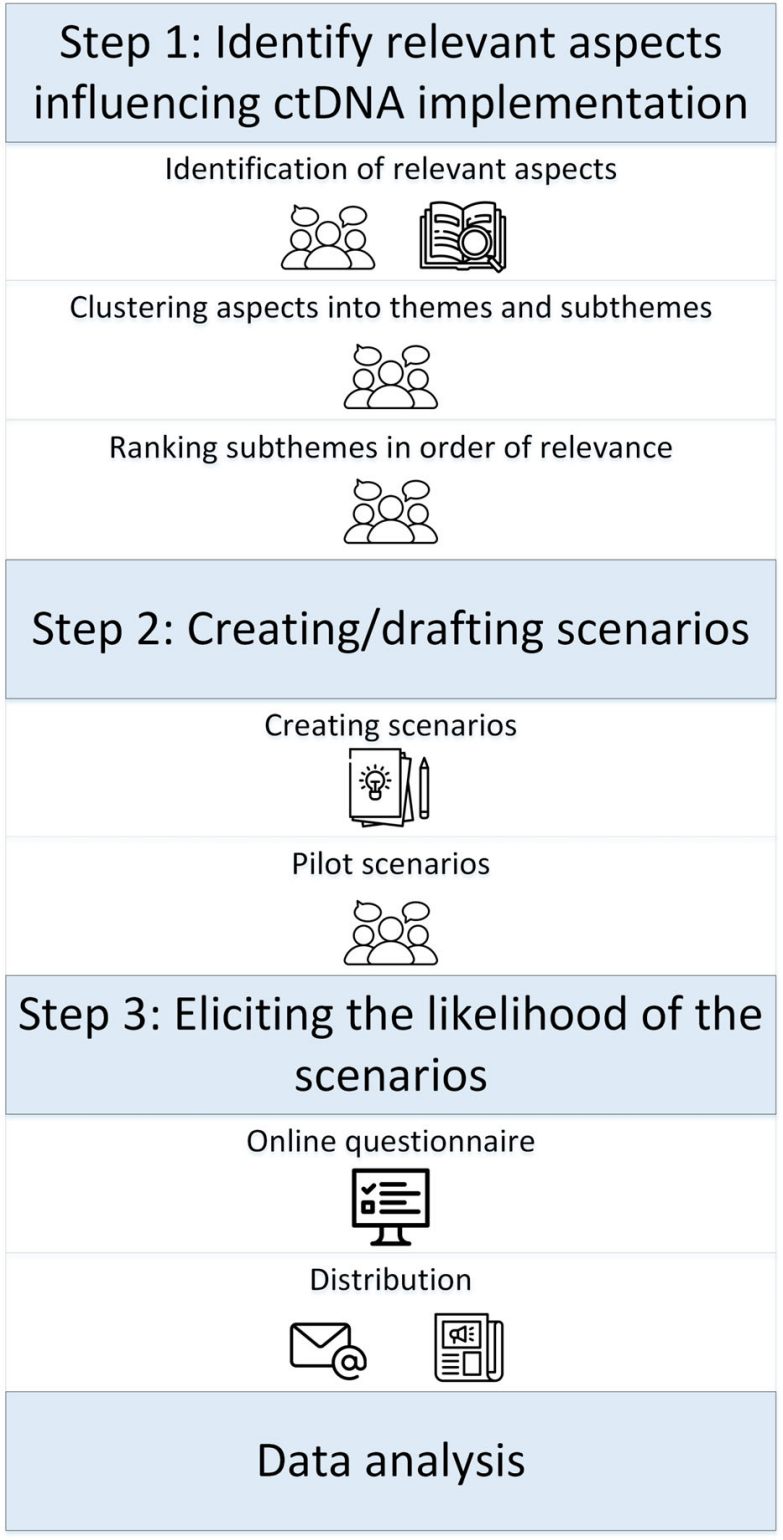
Overview of three‐step study design. Schematic visualization of the three‐step methodology for building and comparing the scenarios.

A scenario‐drafting study was conducted to explore future pathways (i.e. scenarios) of ctDNA testing. This methodology is based on environmental scenario analysis, which is a common practice in environmental science and policy and can also be valuable in health sciences [23, 24, 25, 26]. Scenario drafting can be used to understand and reflect upon the uncertainties about future developments in multiple dimensions, by building potential scenarios, comparing them, and evaluating their impact [23]. This study focuses on the first two features; building and comparing scenarios. It has a three‐step design (Fig. 1): First, relevant aspects that influence the implementation of ctDNA testing were identified. Second, scenarios were drafted based on these aspects. Third, the likelihood of the scenarios was elicited with an online questionnaire among ctDNA experts.

### 2.1 Step 1: Identification of relevant aspects and themes influencing ctDNA implementation

Focus groups were organized for the first step and included experts within the field of translational oncology, laboratory medicine, and health technology assessment who were all actively involved in ctDNA research projects. Background information regarding ctDNA implementation was gathered. First, two researchers (AK, CR) performed a scoping literature review to identify key aspects influencing ctDNA testing implementation (both barriers and facilitators), according to the methodology of Peters et al. [27, 28] (Fig. S1). Secondly, the experts verified that no important aspects were missing during the first focus group. The identified aspects were clustered into themes and subthemes and verified with the experts in a second focus group (Fig. S2). The identified main themes were: clinical utility, economic aspects, organizational aspects, technical aspects, and social aspects. Lastly, the experts ranked the subthemes in order of relevance in a final focus group.

### 2.2 Step 2: Scenario drafting and questionnaire development

The first list of potential scenarios regarding ctDNA testing implementation was drafted: one main scenario about successful implementation, and several scenarios per theme influencing implementation based on the most relevant subthemes found in step 1. To illustrate, an example of a scenario was: “ctDNA testing will be reimbursed within the next five years” (economic subtheme). The scenarios were complemented with additional questions to obtain more context and detailed information about each theme. This first list of scenarios was piloted among eight ctDNA‐experts, and three experts in the field of policy‐making and HTA to evaluate if all scenarios and additional questions included were relevant and uniformly interpreted. The scenarios were adapted according to their feedback.

### 2.3 Step 3: Eliciting the likelihood of the scenarios

#### 2.3.1 Questionnaire

The final questionnaire was incorporated into an online survey tool (Survalyzer Next Generation). The questionnaire included 12 scenarios and 16 additional questions, divided into three parts: (1) evidence generation, (2) successful implementation, and (3) exploring different scenarios per theme (see Table 1). The first part of the questionnaire consisted of two questions to investigate the awareness about the current stage of evidence for the different ctDNA applications and evidence needed to prove clinical utility in the Netherlands, in order to create context for the scenarios proposed in the second and third parts of the questionnaire. The second part of the questionnaire included the main scenario about successful implementation and investigated the general challenges of successful implementation. “Successful implementation” was defined as the ideal situation in which five theme‐specific scenarios are achieved (representing the five themes found in step 1): (1) The test is included in the clinical guidelines (clinical utility), (2) The costs of the test are reimbursed (economical), (3) Analytical procedures for ctDNA analysis are harmonized (pre‐analytical, analytical, reporting) (technical), (4) All logistics are in place so all patients have access to the test (organizational), (5) The test is offered to all patients who can benefit from ctDNA testing (social). The third part included theme‐specific scenarios and questions to explore specific challenges regarding ctDNA implementation. The complete questionnaire can be found in Appendix S1. The likelihood of the scenarios occurring within 5 years was elicited by using a sliding scale from 0% to 100% (0% = will definitely not occur, 100% = will definitely occur). Additional questions consisted of different types of questions (open, dichotomous, multiple choice, rank‐order scaling, and Likert‐scale). Respondents filled in the questionnaire for NSCLC or CRC only, depending on their expertise, and they could skip questions if they did not feel comfortable answering them. When considered necessary, answers were requested per clinical application of ctDNA testing (monitoring treatment response, target profiling, MRD detection, and early detection/screening). The definitions of the applications included in the questionnaire can be found in Table 2.

**Table 1:** Online questionnaire: outline and included themes.

1. Evidence generation
Awareness about the current stage of evidence
Future evidence: Exploring type of evidence needed to prove clinical utility
2. Main implementation scenario
Successful implementation of ctDNA
Challenges regarding successful implementation
3. Theme‐specific scenarios
Clinical utility
Inclusion in clinical guidelines
ctDNA vs current practice
Economical aspects
Cost of test
Reimbursement/funding systems
Organizational aspects
Hospital level
National level
Technical aspects
Analytical aspects
Competition other biomarkers
Social aspects
Health care professionals perspective
Patient perspective

**Table 2:** Definition of the clinical applications of ctDNA testing.

Clinical application ctDNA testing	Definition
Monitoring treatment response	Evaluating the response to treatment over time with serial liquid biopsies to detect disease progression during systemic treatment (chemotherapy, targeted therapy, etc.)
Tumor profiling	Detect specific mutations in clinically‐relevant targets in liquid biopsies with a single test to guide treatment decisions
Minimal residual disease (MRD) detection	Detect presence of ctDNA in liquid biopsies to improve risk stratification and guide adjuvant treatment decisions after treatment with curative intent (e.g. surgery)
Early detection/screening	Detect cancer in liquid biopsies at the earliest possible stage to have the best chance for a successful treatment

#### 2.3.2 Distributing the questionnaire

The target population was experts working in the field of ctDNA testing. The complete questionnaire was distributed via e‐mail and online newsletters from research groups in oncology (Dutch Colorectal Cancer Group; DCCG, and Dutch Oncology Research Platform; DORP) between July and October 2021. Prior to distribution, this study was presented during a COIN consortium meeting in June 2021 to attract respondents. Email recipients included members of the COIN network, members of the researchers' network, and authors of relevant work in the field (127 recipients from 23 Dutch institutes). Recipients were encouraged to forward the questionnaire to their network of ctDNA experts. Reminders were sent to all the recipients of the e‐mail after 2 weeks. All respondents were asked for informed consent before filling in the questionnaire. Respondents who indicated to have no experience in the field of ctDNA testing were excluded from further analysis.

#### 2.3.3 Ethical approval and consent to participate

This study was reviewed and approved by the Institutional Review Board (IRB) from the Netherlands Cancer Institute under number IRBd23‐063. Informed consent was inferred upon completion of the survey and approved by the IRB from the Netherlands Cancer Institute. All methods were carried out in accordance with relevant guidelines.

### 2.4 Step 4: Data analysis

All responses were anonymized. The data were analyzed in Microsoft Excel (version 2016). Descriptive statistics were used for most questions (frequencies and percentages). Median, minimum, and maximum were estimated from the pooled likelihood estimates of the scenarios. Open additional questions were analyzed by inductive coding of the responses [29]. For rank‐order scaling questions, the ranking positions were scored as follows: rank 1 = 5 points, rank 2 = 4 points, rank 3 = 3 points, rank 4 = 2 points, rank 5 = 1 point. Then, weighted averages were calculated by dividing the total sum of the scores of all respondents by the number of respondents, for each answer. Visualization of the data was performed in Microsoft Excel (version 2016) and using r package “ggplot” in rstudio (https://cran.r‐project.org/web/packages/ggplot2/index.html, r version 4.1.2). Missing answers were excluded from the analysis.

## 3 Results

### 3.1 Participant characteristics

Thirty ctDNA‐experts completed the online questionnaire (18 NSCLC‐experts, and 12 CRC‐experts, see Table 3). Most experts had a clinical or technical background and worked in an academic hospital or specialized cancer center, with an average experience with ctDNA testing of 4.9 years. More information about the respondents can be found in Table S1.

**Table 3:** Respondent characteristics.

	*N*
Number of respondents	
NSCLC	18
CRC	12
Profession	
Laboratory specialist	11
Clinical researcher	10
Fundamental researcher	4
Clinical scientist in molecular pathology	3
Policy maker	1
HTA‐researcher/Health economist	1
Place of employment	
Academic hospital	17
Specialized cancer center	9
General hospital	2
Healthcare insurance company	1
University	1
Years of experience with ctDNA	
Mean average	4.9 years

### 3.2 Evidence generation

**Fig. 2:**
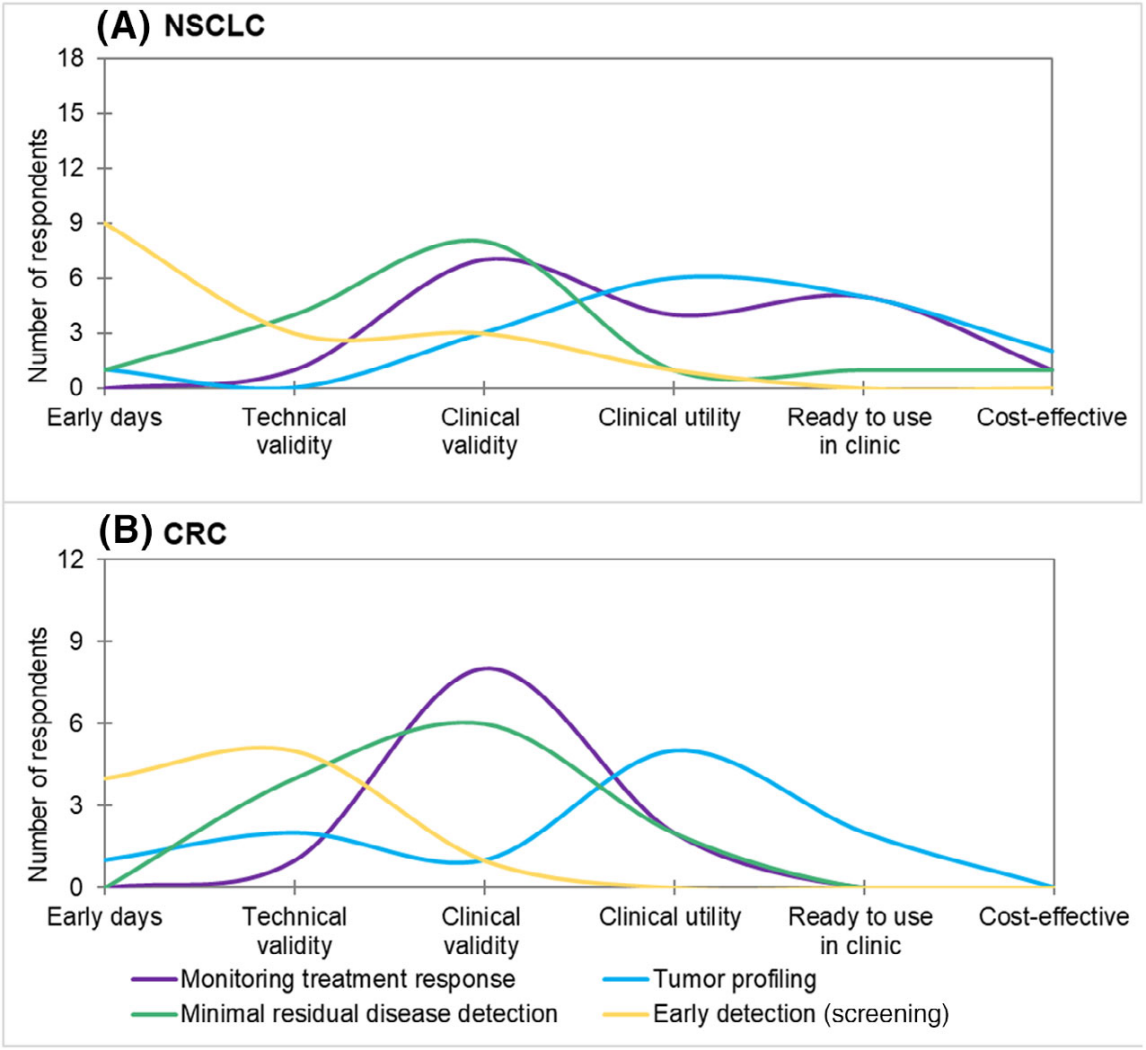
Awareness about the current stage of evidence of the different applications for ctDNA testing in the Netherlands. The *y*‐axis shows the total number of answers is shown, the upper limit of the *y* axis is the total number of respondents per tumor type. In the *x*‐axis shows the stages of evidence [30]. (A) Results for NSCLC. (B) Results for CRC. (1) Early days: new liquid biopsy test is developed. (2) Technical validity: ability to detect and quantify a molecular aberration. (3) Clinical validity: correlation with a clinical outcome such as prognostic value for overall survival. (4) Clinical utility: ability of the liquid biopsy to actually guide treatment decisions that improve clinical outcomes. (5) Ready to use in clinic: level of evidence where clinicians feel the test is ready for use. (6) Cost‐effective: demonstration of an economically viable test relative to the clinical benefit.

The awareness about the current stage of evidence in the implementation process in the Netherlands differs among clinical applications of ctDNA testing and per tumor type (see Fig. 2). Of the four clinical applications, tumor profiling was considered to be at the highest stage of evidence generation in both tumor types, with clinical utility proven for both cancer types, and considered ready to use in the clinic for NSCLC. Monitoring response to treatment was also considered ready for use in the clinic by NSCLC experts. For, MRD detection was at the evidence stage of clinical validity in both cancer types. Early detection/screening was considered to be at the lowest stage of evidence generation for both cancer types, with strong consensus.

Regarding which evidence is necessary to prove clinical utility, the answers differed per application. Notably, the need for large cohort studies was a common answer, while randomized clinical trials (RCTs) were not always considered necessary to prove clinical utility. The suggested endpoints of these large‐cohort studies were both long‐term measures; such as survival benefit for monitoring treatment response and MRD detection, and short‐term measures; such as technical performance and concordance of the test compared to current diagnostic tests for monitoring response to treatment and tumor profiling. For early detection/screening, there was consensus about the need to compare the ctDNA testing approach to the current screening method (e.g. CRC screening program).

### 3.3 Main scenario: successful implementation

**Fig. 3:**
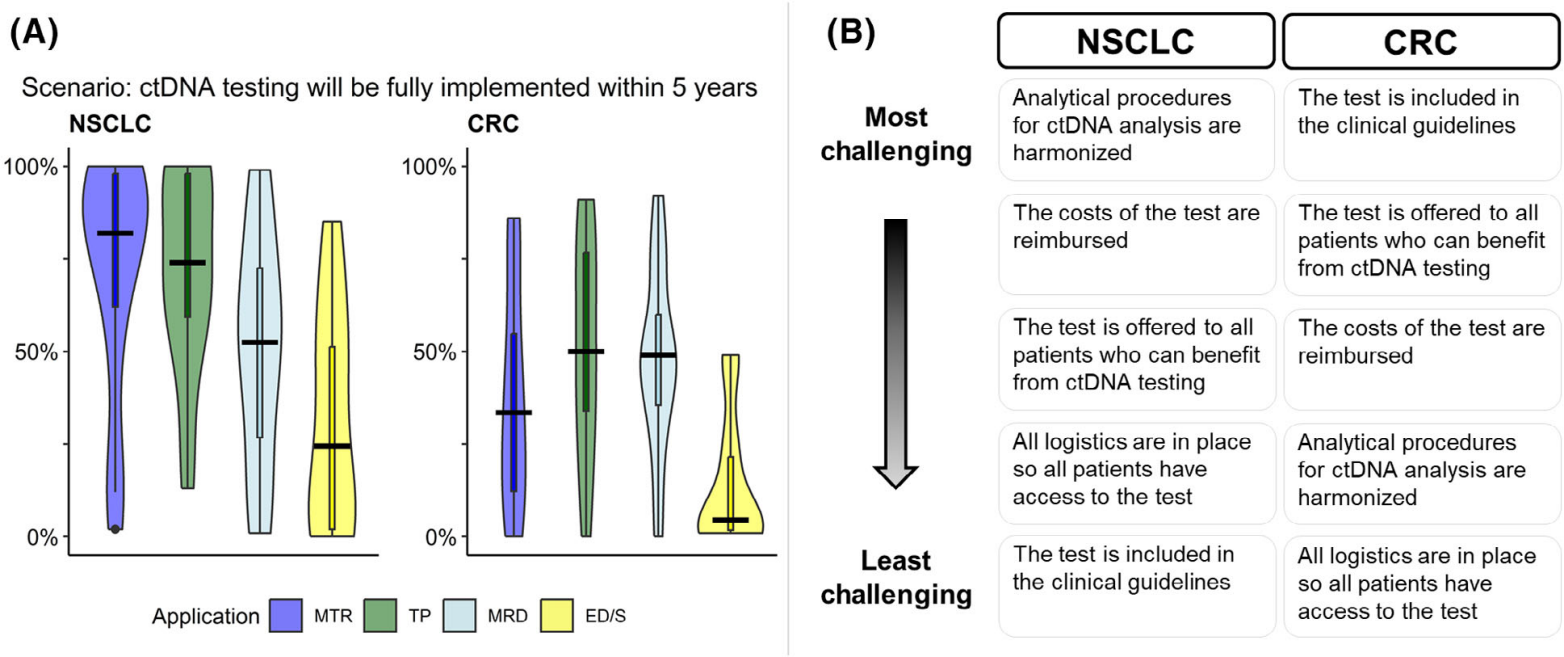
Successful implementation of ctDNA testing: likelihood and main challenges. (A) Results of the scenario per cancer type. *Y*‐axis shows the 5‐year likelihood between 0% and 100% according to the experts. Black line shows the median. (B) Ranking of the challenges to achieve successful implementation. ED/S, early detection/screening; MTR, monitoring treatment response; TP, tumor profiling.

Regarding the main scenario concerning “successful implementation of ctDNA”, NSCLC experts considered tumor profiling and monitoring response to treatment as the respective applications most likely to be successfully implemented in 5 years, with 74% and 82% median likelihoods of achieving the scenario. For all the applications in CRC, the median likelihoods were lower compared to NSCLC and the range of answers was larger. Early detection/screening was seen as the clinical application most distant from successful implementation in both tumor types (NSCLC: 25%, CRC: 4.5% median likelihood) (Fig. 3A and Table S2).

The order of the ranking from most to least challenging to achieve of the five theme‐specific scenarios differed between CRC and NSCLC (Fig. 3B and Fig. S3). While inclusion in the clinical guidelines was seen as the most challenging scenario by CRC experts, it was seen as the least challenging by NSCLC experts. Having the logistics in place was not considered challenging according to both NSCLC and CRC experts (CRC: ranked 5th, NSCLC: ranked 4th).

### 3.4 Theme‐specific scenario's

**Fig. 4:**
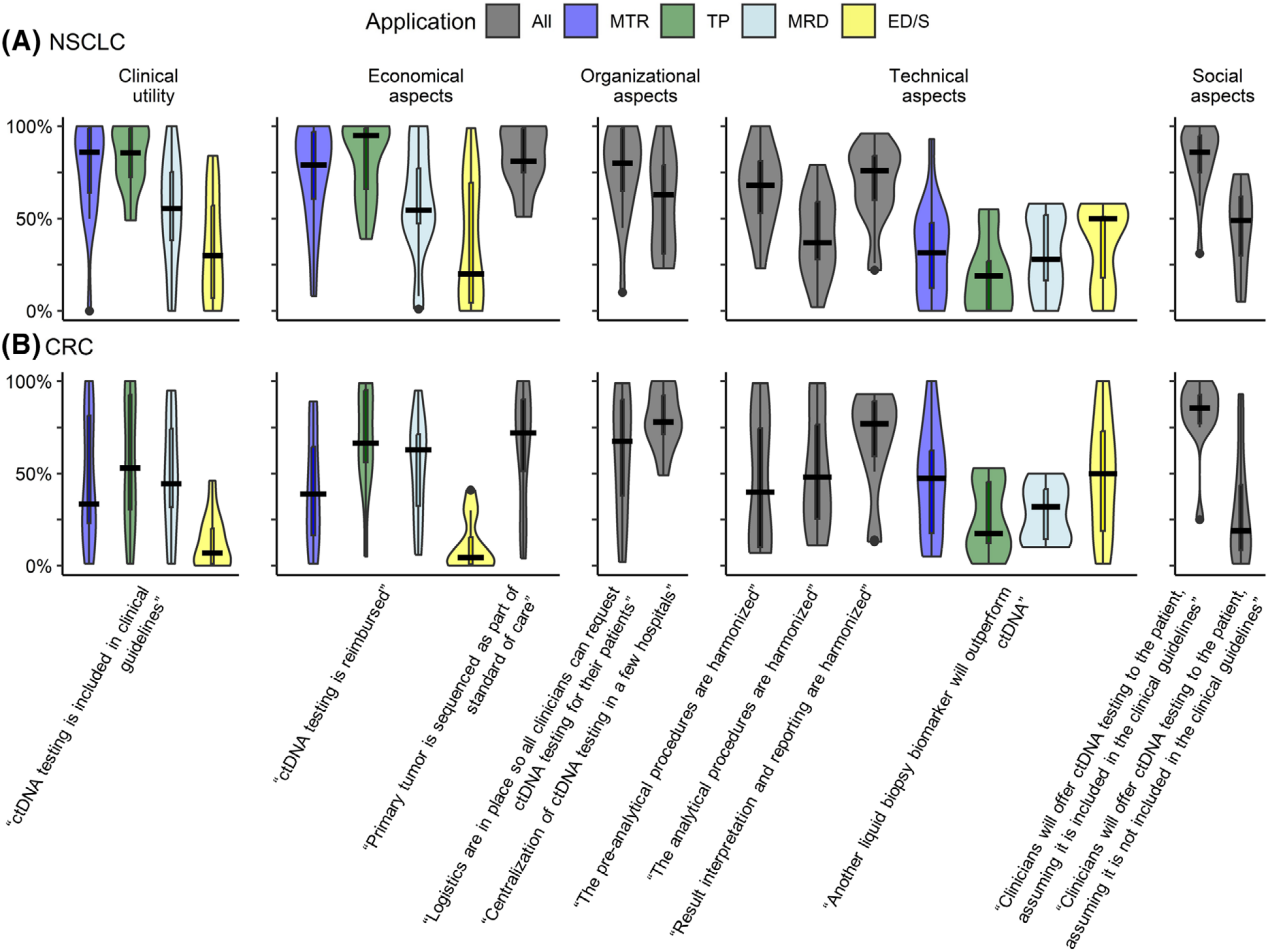
Overview results of 5‐year likelihood per scenario. (A, B) 5‐year likelihood of scenarios regarding ctDNA testing implementation per clinical application. (A) Result for NSCLC. (B) Results for CRC. *X*‐axis shows the scenarios per theme, *y*‐axis shows the likelihood between 0 and 100%. Black line shows the median in each scenario. All, all applications; ED/S, early detection/screening; MTR, monitoring treatment response; TP, tumor profiling.

Estimations of the likelihood of in total 11 theme‐specific scenarios were elicited. An overview of elicited likelihoods and median likelihood per scenario and per cancer type can be found in Fig. 4 and Table S2.

#### 3.4.1 Clinical utility

The median likelihood of the scenario “ctDNA testing will be included in clinical guidelines in the next five years” showed that the respondents considered this scenario most likely to occur for monitoring treatment response and tumor profiling in NSCLC (both 86% median likelihood). In CRC, the answers were widely distributed. In both cancer types, there was consensus about ctDNA testing not being included in guidelines within 5 years for early detection/screening. Additionally, we explored the added value and positioning of ctDNA testing with respect to current standard diagnostic procedures. According to the respondents, ctDNA testing will be used in clinical practice within 5 years along with current diagnostic procedures, and the results will be used in clinical decision‐making (Figs S4 and S5). NSCLC experts deemed that ctDNA testing would significantly improve survival in all applications except early detection/screening, and in CRC this was only indicated for MRD detection (Table S3). Respondents also indicated that the minimally invasive character of liquid biopsies and the possibility of solving an unmet clinical need could also lead to the inclusion of ctDNA testing in guidelines if survival benefit is not yet proven (Table S4).

#### 3.4.2 Economical aspects

The median likelihood of the scenario “ctDNA testing will be reimbursed in the next five years” was highest for tumor profiling and monitoring response to treatment in NSCLC (95% and 79% median likelihood, respectively) and for monitoring response to treatment and MRD detection in CRC (66.5% and 63% median likelihood, respectively). We explored the scenario: “The primary tumor will be sequenced as part of standard of care within five years”, which would limit the costs of ctDNA testing to plasma testing only. Respondents found this scenario likely to occur (NSCLC: 81%, CRC: 72% median likelihood). In addition, most respondents considered it likely that costs of ctDNA testing will decrease or remain stable (63% and 27% of the respondents, respectively) (Fig. S6), and 90% of the respondents with knowledge about the budget for diagnostic procedures indicated that there were currently budget restrictions for ctDNA testing (Fig. S7).

#### 3.4.3 Organizational aspects

The likelihood of the scenario “Logistics are in place so every patient has access to the test in five years” was considered high according to the respondents (median likelihood NSCLC: 81%, CRC: 67.5%). Other suggested requirements to ensure patient access were: reimbursement, education, use of national existing logistics, and the need for communication with hospitals and patients (Table S5). The second organizational scenario “ctDNA testing will be centralized in a few hospitals in the next five years” was deemed likely for most of the respondents, with high consensus for CRC specialists, but a large variety in the opinions among NSCLC experts (median likelihood NSCLC: 57%, CRC: 78%). Cost reduction, increased expertise, and scale efficiencies were seen as the main advantage of centralization, but complex logistics and a possible increase in the turnaround time were mentioned as the main disadvantages (Table S6). Additionally, the possibility of centralization in a single center was considered not helpful for implementation by 80% of the respondents (Fig. S8).

#### 3.4.4 Technical aspects

The likelihood of the scenarios that “ctDNA analysis will be harmonized on a national level in the next five years” for each part of the analysis (pre‐analytical, analytical, and post‐analytical procedures) was explored. Harmonization of post‐analytical procedures (i.e. results interpretation and reporting) was considered likely to occur (median likelihood NSCLC: 76%, CRC: 77%), and harmonization of analytical procedures was considered least likely (median likelihood NSCLC: 37%, CRC: 48%). The likelihood of the scenario that “another liquid biopsy biomarker will outperform ctDNA within five years” was considered unlikely (median likelihoods: 19–50%).

#### 3.4.5 Social aspects

The likelihood of the scenario “clinicians will offer ctDNA testing to the patient within five years” was considered higher if the test is included in the clinical guidelines (NSCLC: 86%, CRC 85.5%) compared to when ctDNA testing is not included in guidelines (NSCLC: 49%, CRC: 19%). Most ctDNA experts agreed that patients would prefer ctDNA testing over current diagnostic methods (Fig. S9).

## 4 Discussion

In this study, we investigated the opinions and expectations of 30 ctDNA‐experts on 12 future scenarios to explore the current status, future developments, and requirements for implementation of ctDNA testing for oncology in the Netherlands. The applications which were considered to have the highest stage of evidence in the implementation process in the Netherlands were also considered more likely to be implemented within 5 years. Early detection/screening was considered least likely to be implemented within this time frame. Based on the results, we identified that demonstrating clinical utility is the main facilitator for ctDNA implementation in Dutch clinical practice; as it could accelerate implementation on multiple levels. However, the type of evidence required to demonstrate clinical utility was unclear and differed per indication and tumor type. We also found that the challenges regarding implementation differed amongst clinical applications and tumor types, for example, inclusion of ctDNA testing in clinical guidelines and centralization of ctDNA analysis. Therefore, to achieve optimal implementation of ctDNA testing in clinical practice, it seems that the implementation process of each clinical application of ctDNA testing should be evaluated per tumor type in context of the clinical application.

The evaluation of diagnostic tests for the implementation in clinical practice has been subject of discussion for many years [6]. The present study confirmed that it is critical to demonstrate clinical utility to achieve successful implementation, in line with the conclusions of a similar study in the Australian setting [30]. In addition, we found that the specific evidence required (i.e. type of study, type of evidence, definition of “sufficient” evidence) is not yet clearly defined for diagnostic tests. For new oncological drugs, the evaluation criteria and endpoints are clearly defined by the Dutch advisory committee for oncological agents “Commissie Beoordeling Oncologische Middelen” (cieBOM) [31]. However, it remains ambiguous which criteria a diagnostic test must meet and which endpoints should be evaluated to demonstrate its clinical utility [7]. Moreover, new drugs need to be investigated in RCTs, but successfully completing an RCT for the investigation of biomarkers is arduous [32]. Our results suggest that evidence from traditional RCTs with long follow‐up (level 1 evidence) is not always considered necessary to demonstrate clinical utility of ctDNA testing, which could save time and costs in evidence generation. Alternatives for RCTs that have been suggested in literature for evaluating the clinical utility of a biomarker are real‐world cohorts, prospective‐retrospective studies, or decision models [32, 33, 34, 35]. Recently, the advisory committee “Commissie Beoordeling Diagnostiek” (cieBOD) was established in the Netherlands to advise on the effectiveness and positioning of new biomarkers to accelerate implementation [36]. This advisory committee could be involved in a deliberative process with all stakeholders (e.g. regulatory bodies, patient advocates, clinicians, etc.) to evaluate if any endpoints besides survival benefits can also be considered in their advice. By defining the specific evidence required to prove clinical utility and the evaluation criteria, clinical studies can be designed more optimally to obtain the type of data needed, which will bring new diagnostic developments to the patient faster.

In general, ctDNA testing was considered more likely to be implemented in NSCLC than in CRC in the next 5 years for all the applications. In addition, we found that CRC experts considered inclusion in clinical guidelines the biggest challenge for successful implementation; even more than ensuring reimbursement, while NSCLC experts consider it least challenging. These differences could be explained by the different clinical needs per tumor type. In NSCLC, there is a clinical need for mutational predictive testing as there are several approved targeted treatments whose administration is based on molecular profiling. As obtaining a tumor tissue biopsy is challenging in ~15% of patients and associated with complications, the use of ctDNA has high clinical need [37]. This promoted the execution of relevant clinical studies with clear endpoints and expedited the inclusion of ctDNA testing in clinical guidelines for metastatic NSCLC, and could explain the more optimistic attitude toward ctDNA testing of NSCLC experts [38, 39, 40, 41]. In contrast, there are currently limited targeted treatment options for CRC, and tumor tissue biopsies are more easily available. Arguably, this resulted in fewer large cohort studies focused on ctDNA analysis and fewer data available for CRC. As the field of personalized medicine evolves, the expectation is that more targeted therapies will become available for CRC as well in the coming years [42], and liquid biopsies testing will also become a minimally invasive option for the new clinical need of profiling the tumor of these patients.

One of the clear differences observed in the results was that CRC experts considered centralization more likely than NSCLC experts. Possibly, this can be explained by the fact that in CRC, blood samples are already collected and stored centrally as part of ongoing substudies of a national cohort study (Prospective Dutch Colorectal Cancer Cohort, PLCRC) [35]. Contrarily, in metastatic NSCLC at least 9 hospitals are already analyzing ctDNA in their diagnostic laboratories for monitoring progression while on targeted therapies and the detection of resistant mechanisms [43]. Centralization of ctDNA testing in a few hospitals regionally in the coming years would facilitate harmonization and would render other advantages mainly derived from scale efficiencies (e.g. lower costs, increased expertise). Even though the most commonly mentioned disadvantage of centralization in this study was that the turnaround time would increase, recent research in Dutch setting has shown that a minimum number of samples needs to be analyzed weekly in a hospital to make the cost per sample acceptable [44]. Currently, the volume per laboratory is low, so regional collaboration would enable reaching the minimum number of samples in a shorter time and the suspected increase in turnaround time due to shipment of the sample would be compensated by the possibility of immediate analysis [19]. In case ctDNA testing is centralized, the analysis and result interpretation could be performed in a central (regional) molecular laboratory with a multi‐disciplinary molecular tumor board [19, 45], and knowledge transfer must be ensured so all hospitals remain involved in the advances in the field, and expertise is not limited to the executing laboratories.

This study also has limitations. First of all, the study was designed as a first step in the implementation process of ctDNA testing to better understand the broader uncertainties, expectations, and potential barriers in multiple themes. Considering our extensive and broad questionnaire, some important topics like the choice of ctDNA testing technology could not be investigated thoroughly within this study. However, the importance of this topic was also recognized within the COIN project, so another work package has been evaluating and publishing on the pre‐analytical and analytical aspects of ctDNA testing in the Netherlands [18]. A second limitation is the timing of the survey. The survey was conducted in 2021, and new evidence have become available on the clinical utility of ctDNA testing, for amongst others for MRD detection in CRC [46, 47]. While the answers to some of the scenarios and questions would be different in case the survey was distributed more recently, we are under the impression that the identified challenges and opportunities still exist and remain a relevant for the implementation of ctDNA testing [39]. Finally, two other important limitations of this study are the number of respondents, and the intrinsic variability that eliciting opinions entails; this resulted in a wide range in the likelihood of the scenarios and the answers to the questions, which reflects the need for a multidisciplinary structured approach toward implementation. Nevertheless, we collected valuable information about views on the current and future status of ctDNA testing. Another point to take into consideration is that, while one strength of this study lies in involving all stakeholders in the development of the questionnaire, most respondents had laboratory background and/or in research. For timely implementation and adoption of ctDNA testing, it is important that the perspectives of all stakeholders are actively participating in future studies from an early stage, such as patient representatives, physicians, and policy‐makers.

## 5 Conclusions

In conclusion, we present a structured description of the opinions of ctDNA experts about potential future pathways for ctDNA testing in the Netherlands. It can be expected that ctDNA testing will continue to be gradually implemented in clinical practice. Challenges related to the rapid advancements in the field, as well as the specific challenges per application and tumor type should be addressed to ensure smooth implementation. The next step toward implementation is to define how clinical utility of biomarkers is demonstrated. This, and other remaining challenges, can be addressed in a deliberative process involving all stakeholders, to ultimately deliver optimal patient care.

## Conflict of interest

CR‐A reports nonfinancial support from Personal Genome Diagnostics and Cergentis BV. DB has provided lectures, expert testimony, and advisory board presence, for Roche diagnostics, all outside the submitted work and all financial supports transferred to institute. GAM is co‐founder and board member (CSO) of CRCbioscreen BV, he has a research collaboration with CZ Health Insurances (cash matching to ZonMW grant), he has research collaborations with Exact Sciences, Sysmex, Sentinel Ch. SpA, Personal Genome Diagnostics (PGDX), DELFi and Hartwig Medical Foundation; these companies provide materials, equipment and/or sample/genomic analyses, and he has several patents pending/issued. GRV reported grants and/or nonfinancial support from BMS, Merck, Servier, Personal Genome Diagnostics, Bayer, Sirtex, Pierre Fabre, Lilly, Delfi Diagnostics, all outside the submitted work and all financial supports transferred to institute. ES provided lectures for Bio‐Rad, Seracare, Roche, Biocartis, Lilly, Agena Bioscience, Illumina, received provided consultation for MSD/Merck, AstraZeneca, Roche, Novartis, Bayer, BMS, Lilly, Amgen, Illumina, Agena Bioscience, CC Diagnostics, Janssen Cilag (Johnson&Johnson), Astellas Pharma, and received research grants from Abbott, Biocartis, Invitae‐ArcherDX, AstraZeneca, Bayer, Bio‐Rad, Roche, Agena Bioscience, CC diagnostics, and Boehringer Ingelheim, all outside the submitted work and all financial supports transferred to institute. RJAF reports grants and nonfinancial support from Personal Genome Diagnostics, DELFI Diagnostics and Cergentis BV; grants from MERCK BV; and nonfinancial support from Pacific Biosciences, outside the submitted work. In addition, R.J.A.F. has several patents pending. VPR received in the past 3 years an unrestricted grant from Intuitive BV, outside of the current work. The remaining authors declare no potential competing interests.

## Author contributions

AK, CR‐A, DB, DCLV, IE, RJAF, VMHC, and VPR are all experts who participated in the focus groups contributed to the conceptualization. AK and CR‐A were involved in methodology, formal analysis, investigation, data curation, writing main manuscript, and visualization. VPR and VMHC were involved in methodology, supervision, project administration, and funding acquisition. DB, GAM, and RJAF were involved in funding acquisition. AK, CR‐A, DB, DCLV, IE, GAM, GRV, ES, RJAF, VMHC, and VPR were involved in interpretation of the data, reviewing and editing the manuscript, and approval of the final version.

### Peer review

The peer review history for this article is available at https://www.webofscience.com/api/gateway/wos/peer‐review/10.1002/1878‐0261.13562.

## Supporting information


**Appendix S1.** Final questionnaire ctDNA testing implementation.

**Fig. S1.** Flowchart scoping literature review.
**Fig. S2.** Identified themes.
**Fig. S3.** Ranking of challenges to achieve successful implementation of ctDNA testing.
**Fig. S4.** Positioning of ctDNA testing in the diagnostic procedures.
**Fig. S5.** Role of ctDNA testing in clinical decision making.
**Fig. S6.** Expectations about cost of ctDNA testing.
**Fig. S7.** Perception about budget restrictions for ctDNA testing.
**Fig. S8.** Opinion about centralization in one center.
**Fig. S9.** Opinion about patient preference for ctDNA testing over current diagnostic methods.

**Table S1.** Detailed respondent characteristics.
**Table S2.** Detailed overview of the results for all scenarios.
**Table S3.** Expectation about obtaining survival benefit as a result of ctDNA testing.
**Table S4.** Aspects different to survival benefit leading to inclusion of ctDNA testing in clinical guidelines.
**Table S5.** Ensuring patient access.
**Table S6.** Main advantages and disadvantages of centralization.

## Data Availability

The questionnaire used is provided as Appendix S1. Data from the answers to the questionnaire are available upon reasonable request from the authors.
