# Supplementary material for: A scenario‐drafting study to explore potential future implementation pathways of circulating tumor DNA testing in oncology
Source: Mol Oncol. 2024 Jan 11;18(11):2730–42. doi: 10.1002/1878-0261.13562 (PMC11547223; doi:10.1002/1878-0261.13562)
Supplement: Supplementary file 1 — Appendix S1. Final questionnaire ctDNA testing implementation. [file MOL2-18-2730-s002.pdf]

SURVALYZER

Survey

FINAL QUESTIONNAIRE CTDNA TESTING IMPLEMENTATION

Table of Contents

Survey ... 1

Introduction and instructions ... 5

Participant characteristics ... 7

q1: What is your profession? ... 7

q2: Where are you employed? ... 7

q3: How many years of experience with ctDNA do you have? ... 7

q4: How much do you know about the following ctDNA applications? ... 7

Evidence generation ... 9

q5: Monitoring treatment response ... 10

q6: Tumor profiling ... 10

q7: Minimal residual disease detection ... 10

q8: Early detection (Screening) ... 10

q9: Monitoring treatment response ... 11

q10: Tumor profiling ... 11

q11: Minimal residual disease detection ... 11

q12: Early detection (Screening) ... 11

Clinical utility ... 12

q13: Monitoring treatment response ... 12

q14: Tumor profiling ... 12

q15: Minimal residual disease detection ... 12

q16: Early detection (Screening) ... 13

q17: Based on your knowledge and opinion, **what will be the positioning of ctDNA testing compared to current diagnostic standards within 5 years for the different applications?** ... 15

q18: **How will the results of ctDNA testing be used to inform clinical decision making within 5 years?** ... 16

q19: **Clinical benefit** Based on your knowledge and opinion, **in which application(s) will ctDNA testing potentially improve survival of the patients significantly?** ... 17

q20: Based on your knowledge and opinion, **if there is no survival benefit: what other aspects of ctDNA testing could lead to its inclusion in clinical guidelines?** ... 17

q21: Based on your knowledge and opinion, **how would ctDNA testing be classified for each application?** ... 18

q22: Based on your knowledge and opinion, **should the level of evidence needed for implementation differ between new diagnostic procedures and further development of existing diagnostic procedures?** 18

Economical aspects 19

q23: Monitoring treatment response 19

q24: Tumor profiling 19

q25: Minimal residual disease detection 19

q26: Early detection (Screening) 20

q27: Based on your knowledge and opinion, **how will the costs of ctDNA testing change within 5 years compared to now?** 20

q28: Based on your knowledge and opinion, **are there currently any budget restrictions for ctDNA testing in the diagnostic setting?** 20

q29: **To enable a shift towards personalized treatments, NGS of the primary tumor might become standard of care. This would facilitate the implementation of tissue-informed ctDNA analysis. Unrelated to ctDNA: Based on your knowledge and opinion, how likely is it that the PRIMARY tumor will be sequenced as part of standard of care within 5 years?** 21

Organizational aspects 22

q30: Based on your knowledge and opinion, **what is the likelihood that centralization of ctDNA testing in a few hospitals (3-5) will occur within 5 years?** 22

q31: Based on your knowledge and opinion, **what would be the main advantage and disadvantage of centralizing the ctDNA testing in few hospitals**? 22

q32: Based on your knowledge and opinion, do you agree with the following statement? **It will help the implementation of ctDNA testing if only ONE center performs all ctDNA testing for all patients in the Netherlands** 23

q33: **Testing availability:** If ctDNA testing is not performed in every hospital, it is important that logistics are in place so also patients from the other hospitals have access to ctDNA testing. Based on your knowledge and opinion, **what is the likelihood that the logistics are in place so all clinicians can request ctDNA testing for their patients within 5 years*?** 23

q34: Based on your knowledge and opinion, **how can we ensure that every patient has access to ctDNA testing?** 23

q35: **For which applications do you think it is likely that private companies will be offering ctDNA testing directly to the patient within 5 years?** 24

Technical aspects 25

q36: For pre-analytical procedures: 25

q37: For analytical procedures: 25

q38: For results interpretation and reporting: 26

q39: Monitoring treatment response 26
q40: Tumor profiling 26
q41: Minimal residual disease detection 26
q42: Early detection (Screening) 26
Social aspects 27
q43: Based on your knowledge and opinion, **what is the likelihood that a clinician in the Netherlands will offer ctDNA testing to the patient within 5 years, assuming it is included in the clinical guidelines?** 27
q44: Based on your knowledge and opinion, **what is the likelihood that a clinician in the Netherlands will offer ctDNA testing to the patient within 5 years, assuming it is NOT included in the clinical guidelines?** 27
q45: **Patients will prefer ctDNA testing** 29
Full implementation 30
q46: Based on your knowledge and opinion, **rank these 5 tracks from MOST challenging to achieve to LEAST challenging to achieve.** 30
q47: Monitoring treatment response 31
q48: Tumor profiling 31
q49: Minimal residual disease detection 31
q50: Early detection (Screening) 31
Closing 32
q51: If you want to leave any final remarks, use the text box below: 32
Code Plan .... **Error! Bookmark not defined.**

Introduction and instructions

Study aim:
Circulating tumor DNA (ctDNA) has emerged as a promising biomarker with multiple potential applications in cancer care.

This questionnaire is part of a study within the COIN-project, which aims to unite all relevant disciplines to take the essential steps for the implementation of circulating tumor DNA (ctDNA) testing in liquid biopsies in a controlled, cost-effective and validated manner in clinical practice in the Netherlands. For this purpose, we want to explore possible future pathways for the implementation of ctDNA testing, using scenario-drafting.

We are interested in your view about the future of ctDNA testing from your field of expertise.

Explanation of the questionnaire:
To prepare and structure this scenario-drafting study, a scoping literature review was conducted. Aspects influencing the implementation of ctDNA were identified and organized into 6 domains: evidence generation, clinical utility, economical aspects, technical aspects, organizational aspects and social aspects. The most important aspects per domain have been included in this questionnaire.

The questionnaire consists of 7 parts: (1-6) the domains of the scoping litature review + (7) full implementation.

Each part consists of scenarios for which we ask the likelihood of it happening within 5 years **in the Netherlands**, and follow up questions. For some questions, answers per application of ctDNA are asked.

In this questionnaire, we are interested in the following four applications:

	Application	Role of ctDNA testing in blood/liquid biopsies
1	**Monitoring treatment response**	Evaluating the response to treatment over time with serial liquid biopsies to detect disease progression during systemic treatment (chemotherapy, targeted therapy, etc.).
2	**Tumor profiling**	Detect specific mutations in liquid biopsies with a single test to guide treatment decisions.
3	**Minimal residual disease detection after surgery**	Detect presence of ctDNA in liquid biopsies to improve risk stratification and guide adjuvant treatment decisions after surgery.
4	**Early detection (Screening)**	Detect cancer in liquid biopsies at the earliest possible stage to have the best chance for a successful treatment.

All answers will be treated anonymously. Please try to answer all questions to the best of your knowledge, but if you don't feel comfortable answering them, it is possible to skip questions by answering "prefer not to respond".

Thank you for your time, contribution and effort!

Abbreviations used:
PCR: Polymerase Chain Reaction
NGS: Next Generation Sequencing
WGS: Whole Genome Sequencing

Participant characteristics

Respondent characteristics

q1: What is your profession?

- ○ Clinical researcher
- ○ Fundamental researcher
- ○ Laboratory specialist
- ○ Clinician/Medical doctor (no research)
- ○ Policy maker
- ○ HTA-researcher/Health economist
- ○ Other: ___________________________

q2: Where are you employed?

- ○ Academic hospital
- ○ General hospital
- ○ Specialized cancer center
- ○ Government organization
- ○ Health insurance company
- ○ Pharmaceutical company
- ○ University
- ○ Other: ___________________________

q3: How many years of experience with ctDNA do you have?

___________________________ *(validation: floating point number)*

q4: How much do you know about the following ctDNA applications?

	No knowledge	Basic knowledge	Expert knowledge
Monitoring treatment response	○	○	○

Tumor profiling	○	○	○
Minimal residual disease detection after surgery	○	○	○
Early detection (Screening)	○	○	○

Evidence generation

1. Evidence generation

Reminder: please fill in the questionnaire for (NSCLC/CRC)

In the table below you can find an overview and explanation of the different applications of ctDNA that are included in this questionnaire. For some questions, it is necessary to make the comparison to the current standard of care. This is different for the different applications, and can also be found in the table below.

Table 1: ctDNA applications, description and current standard of care:

	Application	Role of ctDNA testing in blood/liquid biopsies	Standard of care
1	**Monitoring treatment response**	Evaluating the response to treatment over time with serial liquid biopsies to detect disease progression during systemic treatment (chemotherapy, targeted therapy, etc.).	Evaluating response to treatment with serial radiological evaluations/imaging.
2	**Tumor profiling**	Detect specific mutations in liquid biopsies with a single test to guide treatment decisions.	Biomarkers detection in tissue biopsy to guide treatment decisions (mutations, immunohistochemistry, FISH, etc.)
3	**Minimal residual disease detection after surgery**	Detect presence of ctDNA in liquid biopsies to improve risk stratification and guide adjuvant treatment decisions after surgery.	Adjuvant treatment decisions after surgery based mostly in clinicopathological characteristics (TNM driven).
4	**Early detection (Screening)**	Detect cancer in liquid biopsies at the earliest possible stage to have the best chance for a successful treatment.	For some cancer types, national screening programs (FIT-test/colonoscopy for colorectal cancer, mammography for breast cancer, etc.)

The levels of evidence for the use of ctDNA in practice differ between applications of ctDNA and cancer types.

Please use the slider to indicate what the level of evidence is for the different applications in non-small cell lung cancer, based on your knowledge and opinion.

Background information:
the levels of evidence defined by (Ijzerman et al, 2021):

- **Early days**: new liquid biopsy test is developed.
- **Technical validity**: ability to detect and quantify a molecular aberration.
- **Clinical validity**: correlation with a clinical outcome such as prognostic value for overall survival.
- **Clinical utility**: ability of the liquid biopsy to actually guide treatment decisions that improve clinical outcomes.
- **Ready to use in clinic**: level of evidence where clinicians feel the test is ready for use
- **Cost-effective**: demonstration of an economically viable test relative to the clinical benefit.

q5: Monitoring treatment response (Slider)
Min: 1 - Early daysMax: 6 - Cost-effective

Prefer not to respond

q6: Tumor profiling (Slider)
Min: 1 - Early daysMax: 6 - Cost-effective

Prefer not to respond

q7: Minimal residual disease detection (Slider)
Min: 1 - Early daysMax: 6 - Cost-effective

Prefer not to respond

q8: Early detection (Screening) (Slider)
Min: 1 - Early daysMax: 6 - Cost-effective

Prefer not to respond

Based on your knowledge and opinion, **what type of evidence is needed to prove clinical utility of ctDNA testing for the different applications:**

For example: type of study/trial, endpoint to be proven, etc.

q9: Monitoring treatment response (Open answer)

____

Prefer not to respond

q10: Tumor profiling (Open answer)
For example: type of study/trial, endpoint to be proven, etc.

____

Prefer not to respond

q11: Minimal residual disease detection (Open answer)
For example: type of study/trial, endpoint to be proven, etc.

____

Prefer not to respond

q12: Early detection (Screening) (Open answer)
For example: type of study/trial, endpoint to be proven, etc.

____

Prefer not to respond

2. Clinical utility

Reminder: please fill in the questionnaire for (NSCLC/CRC)

Clinical guidelines

Based on your knowledge and opinion, **how likely is it that ctDNA testing will be included in clinical guidelines within 5 years for the different applications?**

Background information:
Currently, the SONCOS (stichting oncologische samenwerking) has taken control over the development of the clinical guidelines in oncology. The Dutch clinical guidelines for oncology are published on www.richtlijnendatabase.nl.

q13: Monitoring treatment response (Slider)
0% = "Very unlikely to happen"
100% = "Very likely to happen"

Min: 0 - 0Max: 100 - 100

Prefer not to respond

q14: Tumor profiling (Slider)
0% = "Very unlikely to happen"
100% = "Very likely to happen"

Min: 0 - 0Max: 100 - 100

Prefer not to respond

q15: Minimal residual disease detection (Slider)
0% = "Very unlikely to happen"
100% = "Very likely to happen"

Min: 0 - 0Max: 100 - 100

Prefer not to respond

q16: Early detection (Screening) (Slider)

0% = "Very unlikely to happen"
100% = "Very likely to happen"

Min: 0 - 0Max: 100 - 100

Prefer not to respond

Inform clinical decision making

There are diagnostic tests currently in place and being used to guide treatment decisions. (See Table 1 again as a reminder of current standard of care).

Table 1: ctDNA applications, definition and current standard of care:

	Application	Role of ctDNA testing in blood/liquid biopsies	Standard of care
**1**	**Monitoring treatment response**	Evaluating the response to treatment over time with serial liquid biopsies to detect disease progression during systemic treatment (chemotherapy, targeted therapy, etc.).	Evaluating response to treatment with serial radiological evaluations/imaging.
**2**	**Tumor profiling**	Detect specific mutations in liquid biopsies with a single test to guide treatment decisions.	Biomarkers detection in tissue biopsy to guide treatment decisions (mutations, immunohistochemistry, FISH, etc.)
**3**	**Minimal residual disease detection after surgery**	Detect presence of ctDNA in liquid biopsies to improve risk stratification and guide adjuvant treatment decisions after surgery.	Adjuvant treatment decisions after surgery based mostly in clinicopathological characteristics (TNM driven).
**4**	**Early detection (Screening)**	Detect cancer in liquid biopsies at the earliest possible stage to have the best chance for a successful treatment.	For some cancer types, national screening programs (FIT-test/ colonoscopy for colorectal cancer, mammography for breast cancer, etc.)

Therefore, the positioning and the added value of ctDNA testing in cancer care needs to be assessed compared to the current standard diagnostic method.

q17: Based on your knowledge and opinion, **what will be the positioning of ctDNA testing compared to current diagnostic standards within 5 years for the different applications?**

More than 1 answer possible.
Try to include all factors than can influence this decision.

	ctDNA testing will replace current diagnostic methods.	ctDNA testing will be used simultaneously/as a complement of current diagnostic methods.	ctDNA testing will only be used when current diagnostic methods are inconclusive/fail.	ctDNA testing will not be used in clinical practice.	Prefer not to respond
Monitor treatment response	▪	▪	▪	▪	▪
Tumor profiling	▪	▪	▪	▪	▪
Minimal residual disease detection	▪	▪	▪	▪	▪
Early detection (Screening)	▪	▪	▪	▪	▪

q18: **How will the results of ctDNA testing be used to inform clinical decision making within 5 years?**

More than 1 answer possible.
Try to include all factors than can influence this decision.

	Clinical decision making will only be based on ctDNA testing result.	Clinical decision making will only be based on current standard diagnostic methods.	Clinical decision making will be **based on both** the results of standard diagnostic methods and ctDNA testing, but ctDNA testing results are leading in case of contradictory results.	Clinical decision making will be **based on both** the results of standard diagnostic methods and ctDNA testing, but results of standard diagnostic methods are leading in case of contradictory results	Prefer not to respond
Monitor treatment response	▪	▪	▪	▪	▪
Tumor profiling	▪	▪	▪	▪	▪
Minimal residual disease detection	▪	▪	▪	▪	▪
Early detection (Screening)	▪	▪	▪	▪	▪

q19: **Clinical benefit**
Based on your knowledge and opinion, **in which application(s) will ctDNA testing potentially improve survival of the patients significantly?**

More than 1 answer possible.
Survival = Overall survival, Relapse Free survival, Progression Free survival)

- Monitoring treatment response
- Tumor profiling
- Minimal residual disease detection
- Early detection (Screening)
- None of them
- Prefer not to respond

q20: Based on your knowledge and opinion, **if there is no survival benefit: what other aspects of ctDNA testing could lead to its inclusion in clinical guidelines?**

More than 1 answer possible.
Survival = Overall survival, Relapse Free survival or Progression Free survival

- Unmet clinical need
- High burden of tissue biopsy methods (liquid biopsies ess invasive)
- High concordance with current diagnostics methods
- Improvement of Quality of life
- Other: ___________________________
- Prefer not to respond

q21: Based on your knowledge and opinion, **how would ctDNA testing be classified for each application?**

Background information:
When evaluating new molecular diagnostic methods, a distinction could be made between actual new diagnostic methods and further development of existing diagnostic methods.
For example: EGFR mutation detection in metastatic lung cancer was already done in tissue, so ctDNA testing for EGFR mutations detection could be seen as an evolution of an existing diagnostic method.

	New diagnostic procedure	Further development of an existing diagnostic procedure	Prefer not to respond
Monitoring treatment response	○	○	○
Tumor profiling	○	○	○
Minimal residual disease detection after surgery	○	○	○
Early detection (Screening)	○	○	○

q22: Based on your knowledge and opinion, **should the level of evidence needed for implementation differ between new diagnostic procedures and further development of existing diagnostic procedures?**

Background information:
When evaluating new molecular diagnostic methods, a distinction could be made between actual new diagnostic methods and further development of existing diagnostic methods.
For example: EGFR mutation detection in metastatic lung cancer was already done in tissue, so ctDNA testing for EGFR mutations detection could be seen as an evolution of an existing diagnostic method.

- ○ Yes
- ○ No
- ○ Prefer not to respond

Economical aspects

3. Economical aspects

Reminder: please fill in the questionnaire for (NSCLC/CRC)

For the implementation of ctDNA testing, information about the costs and economic evaluations (e.g. cost-effectiveness analysis) are important and are used to inform reimbursement decisions.

Reimbursement

Based on your knowledge and opinion, **what is the likelihood that ctDNA testing will be reimbursed within 5 years for the different applications?**

q23: Monitoring treatment response (Slider)
0% = "Very unlikely to happen"
100% = "Very likely to happen"

Min: 0 - 0Max: 100 - 100

Prefer not to respond

q24: Tumor profiling (Slider)
0% = "Very unlikely to happen"
100% = "Very likely to happen"

Min: 0 - 0Max: 100 - 100

Prefer not to respond

q25: Minimal residual disease detection (Slider)
0% = "Very unlikely to happen"
100% = "Very likely to happen"

Min: 0 - 0Max: 100 - 100

Prefer not to respond

q26: Early detection (Screening) (Slider)
0% = "Very unlikely to happen"
100% = "Very likely to happen"

Min: 0 - 0Max: 100 - 100

Prefer not to respond

Costs of the test

Little is known about the exact costs of ctDNA testing. The cost of ctDNA testing is influenced by many factors, including the platform that is used (e.g. PCR or NGS), the number of genes analyzed, and the number of samples that are analyzed. Therefore, the cost can range from €50 up to thousands of euros per sample.

q27: Based on your knowledge and opinion, **how will the costs of ctDNA testing change within 5 years compared to now?**

Background information:
Costs of a new technology can be influenced by many price mechanisms. For example:

- *costs can decrease because there is more competition*
- *costs can increase because technology becomes more advanced and expensive*
- *costs can decrease because technology becomes more advanced, but also more efficient.*

(Slider)

Min: 1 - Costs will decrease a lotMax: 5 - Costs will increase a lot

Prefer not to respond

q28: Based on your knowledge and opinion, **are there currently any budget restrictions for ctDNA testing in the diagnostic setting?**

- Yes
- No
- Prefer not to respond

q29: **To enable a shift towards personalized treatments, NGS of the primary tumor might become standard of care. This would facilitate the implementation of tissue-informed ctDNA analysis.**

Unrelated to ctDNA:

Based on your knowledge and opinion, <u>how likely is it that the PRIMARY tumor will be sequenced as part of standard of care within 5 years?</u>

(Slider)

0% = "Very unlikely to happen"
100% = "Very likely to happen"

Min: 0 - 0Max: 100 - 100

Prefer not to respond

Organizational aspects

4. Organizational aspects

Reminder: please fill in the questionnaire for (NSCLC/CRC)

Centralization

There seems to be a trend for centralization of complex molecular diagnostics, like NGS, which could also be applicable to the implementation of ctDNA testing: only few hospitals in the Netherlands will perform ctDNA testing, and the other hospitals will send the samples to them.

q30: Based on your knowledge and opinion, **what is the likelihood that centralization of ctDNA testing in a few hospitals (3-5) will occur within 5 years?**
(Slider)
0% = "Very unlikely to happen"
100% = "Very likely to happen"

Min: 0 - 0Max: 100 - 100

Prefer not to respond

q31: Based on your knowledge and opinion, **what would be the main advantage and disadvantage of centralizing the ctDNA testing in few hospitals**? (Open answer)

Prefer not to respond

q32: Based on your knowledge and opinion, do you agree with the following statement? **It will help the implementation of ctDNA testing if only ONE center performs all ctDNA testing for all patients in the Netherlands**

Example: For WGS this is currently done by the Hartwig Medial Foundation (HMF).

(Slider)

Min: 1 - Completely disagreeMax: 5 - Completely agree

Prefer not to respond

q33: **Testing availability:**
If ctDNA testing is not performed in every hospital, it is important that logistics are in place so also patients from the other hospitals have access to ctDNA testing.

Based on your knowledge and opinion, **what is the likelihood that the logistics are in place so all clinicians can request ctDNA testing for their patients within 5 years*?**

**For the applications that have been included in the clinical guidelines*

(Slider)
0% = "Very unlikely to happen"
100% = "Very likely to happen"

Min: 0 - 0Max: 100 - 100

Prefer not to respond

q34: Based on your knowledge and opinion, **how can we ensure that every patient has access to ctDNA testing?**

Prefer not to respond

q35: **For which applications do you think it is likely that private companies will be offering ctDNA testing directly to the patient within 5 years?**

	Unlikely	Neutral	Likely	Prefer not to respond
Monitoring treatment response	○	○	○	○
Tumor profiling	○	○	○	○
Minimal residual disease detection	○	○	○	○
Early detection (Screening)	○	○	○	○

Technical aspects

5. Technical aspects

Reminder: please fill in the questionnaire for (NSCLC/CRC)

Harmonization

The number of techniques and platforms for ctDNA detection is rapidly growing and evolving during the recent years. Differences in the pre-analytical, analytical and post-analytical steps, and laboratory settings can lead to differences in the results of the test. Therefore, technical harmonization and quality assurance are important for the use of ctDNA testing to achieve the same results and/or interpretation of the results when using different tests, or when doing the same ctDNA test in different laboratories.

Based on your knowledge and opinion, **what is the likelihood that ctDNA analysis is harmonized on a national level within 5 years for each part of the analysis (pre-analytical, analytical and results reporting)?**

q36: For pre-analytical procedures: (Slider)
0% = "Very unlikely to happen"
100% = "Very likely to happen"

Min: 0 - 0Max: 100 - 100

Prefer not to respond

q37: For analytical procedures: (Slider)
0% = "Very unlikely to happen"
100% = "Very likely to happen"

Min: 0 - 0Max: 100 - 100

Prefer not to respond

q38: For results interpretation and reporting: (Slider)
0% = "Very unlikely to happen"
100% = "Very likely to happen"

Min: 0 - 0Max: 100 - 100

Prefer not to respond

Based on your knowledge and opinion, **how likely is it that another liquid biopsy biomarker will outperform ctDNA within 5 years per application?**

q39: Monitoring treatment response (Slider)
0% = "Very unlikely to happen"
100% = "Very likely to happen"

Min: 0 - 0Max: 100 - 100

Prefer not to respond

q40: Tumor profiling (Slider)
0% = "Very unlikely to happen"
100% = "Very likely to happen"

Min: 0 - 0Max: 100 - 100

Prefer not to respond

q41: Minimal residual disease detection (Slider)
0% = "Very unlikely to happen"
100% = "Very likely to happen"

Min: 0 - 0Max: 100 - 100

Prefer not to respond

q42: Early detection (Screening) (Slider)
0% = "Very unlikely to happen"
100% = "Very likely to happen"

Min: 0 - 0Max: 100 - 100

Prefer not to respond

6. Social aspects

Reminder: please fill in the questionnaire for (NSCLC/CRC)

When studying implementation, the social domain cannot be overlooked. Topics as education of current and future stakeholders, adoption of the technology and patient view are also important.

Clinicians:

q43: Based on your knowledge and opinion, **what is the likelihood that a clinician in the Netherlands will offer ctDNA testing to the patient within 5 years, assuming it is included in the clinical guidelines?**

So clinicians are aware of the inclusion of ctDNA testing in clinical guidelines AND are willing to offer it to their patients.

(Slider)
0% = "Very unlikely to happen"
100% = "Very likely to happen"

Min: 0 - 0Max: 100 - 100

Prefer not to respond

q44: Based on your knowledge and opinion, **what is the likelihood that a clinician in the Netherlands will offer ctDNA testing to the patient within 5 years, assuming it is NOT included in the clinical guidelines?**

(Slider)
0% = "Very unlikely to happen"
100% = "Very likely to happen"

Min: 0 - 0Max: 100 - 100

Prefer not to respond

Patients:

Based on your knowledge and opinion, do you agree with the following statement:
Most patients will prefer ctDNA testing over current standard diagnostics if both are offered, assuming that they both have a sufficient performance level.

The table below shows again the current standard.
Table 1: ctDNA applications, definition and current standard of care:

	Application	Role of ctDNA testing in blood/liquid biopsies	Standard of care
1	**Monitoring treatment response.**	Evaluating the response to treatment over time with serial liquid biopsies to detect disease progression during systemic treatment (chemotherapy, targeted therapy, etc.).	Evaluating response to treatment with serial radiological evaluations/imaging.
2	**Tumor profiling**	Detect specific mutations in liquid biopsies with a single test to guide treatment decisions.	Biomarkers detection in tissue biopsy to guide treatment decisions (mutations, immunohistochemistry, FISH, etc.)
3	**Minimal residual disease detection after surgery**	Detect presence of ctDNA in liquid biopsies to improve risk stratification and guide adjuvant treatment decisions after surgery.	Adjuvant treatment decisions after surgery based mostly in clinicopathological characteristics (TNM driven).
4	**Early detection (Screening)**	Detect cancer in liquid biopsies at the earliest possible stage to have the best chance for a successful treatment.	For some cancer types, national screening programs (FIT-test/ colonoscopy for colorectal cancer, mammography for breast cancer, etc.)

q45: **Patients will prefer ctDNA testing**

	Completely disagree	Disagree	Not agree / not disagree	Agree	Completely agree	Prefer not to respond
Monitoring treatment response	○	○	○	○	○	○
Tumor profiling	○	○	○	○	○	○
Minimal residual disease detection	○	○	○	○	○	○
Early detection (Screening)	○	○	○	○	○	○
	○	○	○	○	○	○

Full implementation

7. Full implementation

Reminder: please fill in the questionnaire for (NSCLC/CRC)

As we have discussed throughout the questionnaire, the implementation process consists of several interconnected tracks. When all of them are completed, full implementation will be achieved.
Ideally, we would consider full implementation when all of these 5 tracks are completed:

1. The test is included in the clinical guidelines.
2. The costs of the test are reimbursed.
3. Analytical procedures for ctDNA analysis are harmonized (pre-analytical, analytical, reporting).
4. All logistics are in place so all patients have access to the test.
5. The test is offered to all patients who can benefit from ctDNA testing.

q46: Based on your knowledge and opinion, **rank these 5 tracks from MOST challenging to achieve to LEAST challenging to achieve.**

The test is included in the clinical guidelines.
The costs of the test are reimbursed
Analytical procedures for ctDNA analysis are harmonized
All logistics are in place so all patients have access to the test
The test is offered to all patients who can benefit from ctDNA testing

Considering this definition of **<u>full</u>** implementation and based on your knowledge and opinion, **<u>what is the likelihood that ctDNA testing will be implemented within 5 years for the different applications?</u>**

q47: Monitoring treatment response (Slider)
0% = "Very unlikely to happen"
100% = "Very likely to happen"

Min: 0 - 0Max: 100 - 100

Prefer not to respond

q48: Tumor profiling (Slider)
0% = "Very unlikely to happen"
100% = "Very likely to happen"

Min: 0 - 0Max: 100 - 100

Prefer not to respond

q49: Minimal residual disease detection (Slider)
0% = "Very unlikely to happen"
100% = "Very likely to happen"

Min: 0 - 0Max: 100 - 100

Prefer not to respond

q50: Early detection (Screening) (Slider)
0% = "Very unlikely to happen"
100% = "Very likely to happen"

Min: 0 - 0Max: 100 - 100

Prefer not to respond

Closing

Thank you very much for your participation!
Please also forward this questionnaire to your colleagues.

If you have a question and/or want to be informed of the results of the questionnaire and receive the final publication, please send an email to coin@nki.nl.

q51: If you want to leave any final remarks, use the text box below:

Thank you, your answers were saved perfectly.
