# Supplementary material for: A scenario‐drafting study to explore potential future implementation pathways of circulating tumor DNA testing in oncology
Source: Mol Oncol. 2024 Jan 11;18(11):2730–42. doi: 10.1002/1878-0261.13562 (PMC11547223; doi:10.1002/1878-0261.13562)
Supplement: Supplementary file 2 — Fig. S1. Flowchart scoping literature review. Fig. S2. Identified themes. Fig. S3. Ranking of challenges to achieve successful implementation of ctDNA testing. Fig. S4. Positioning of ctDNA testing in the diagnostic procedures. Fig. S5. Role of ctDNA testing in clinical decision making. Fig. S6. Expectations about cost of ctDNA testing. Fig. S7. Perception about budget restrictions for ctDNA testing. Fig. S8. Opinion about centralization in one center. Fig. S9. Opinion about patient preference for ctDNA testing over current diagnostic methods. [file MOL2-18-2730-s003.pdf]

Supplementary figure 1. Flowchart scoping literature review.

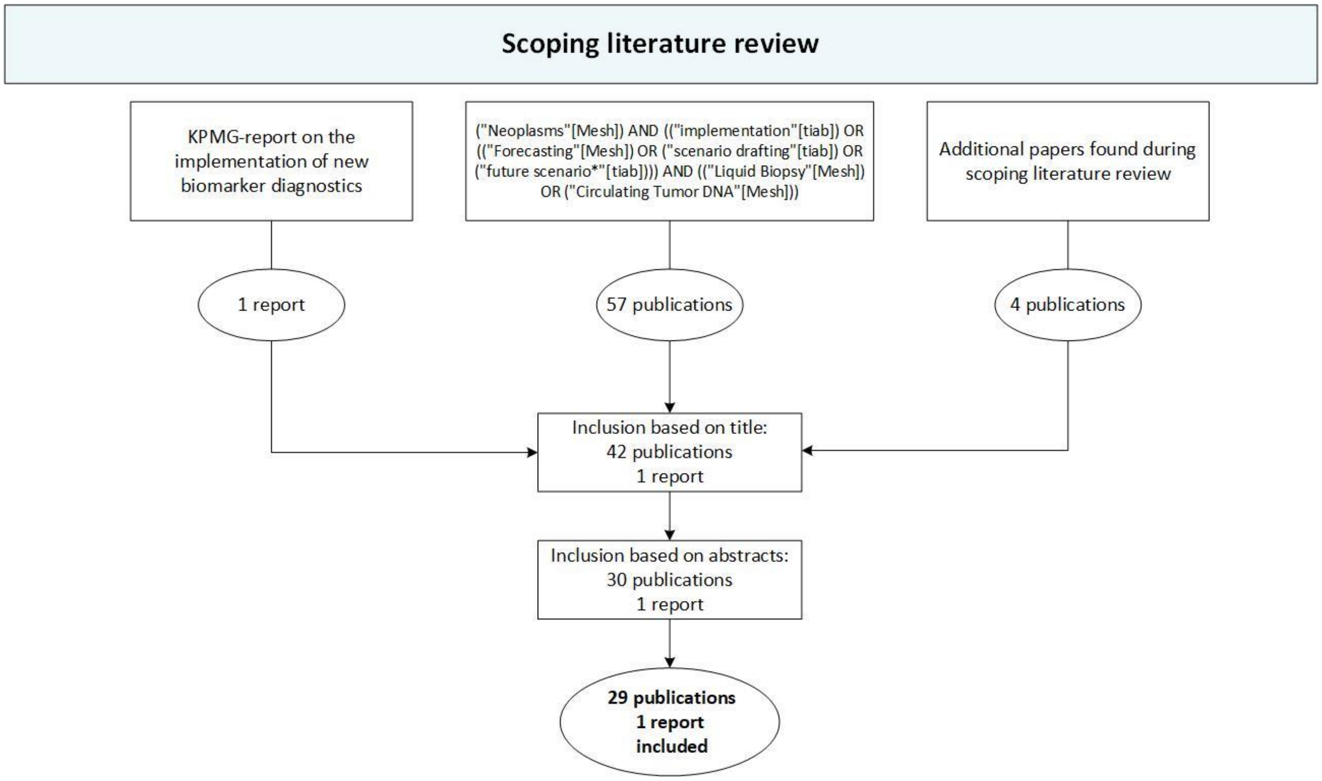

Supplementary figure 1: The literature search was performed in PubMed. The first 10 articles were evaluated independently by both researchers and the data extracted was discussed to ensure consistency in the study selection/inclusion process. The remaining articles were evaluated by one of the researchers and discussed together.

Supplementary figure 2. Identified themes.

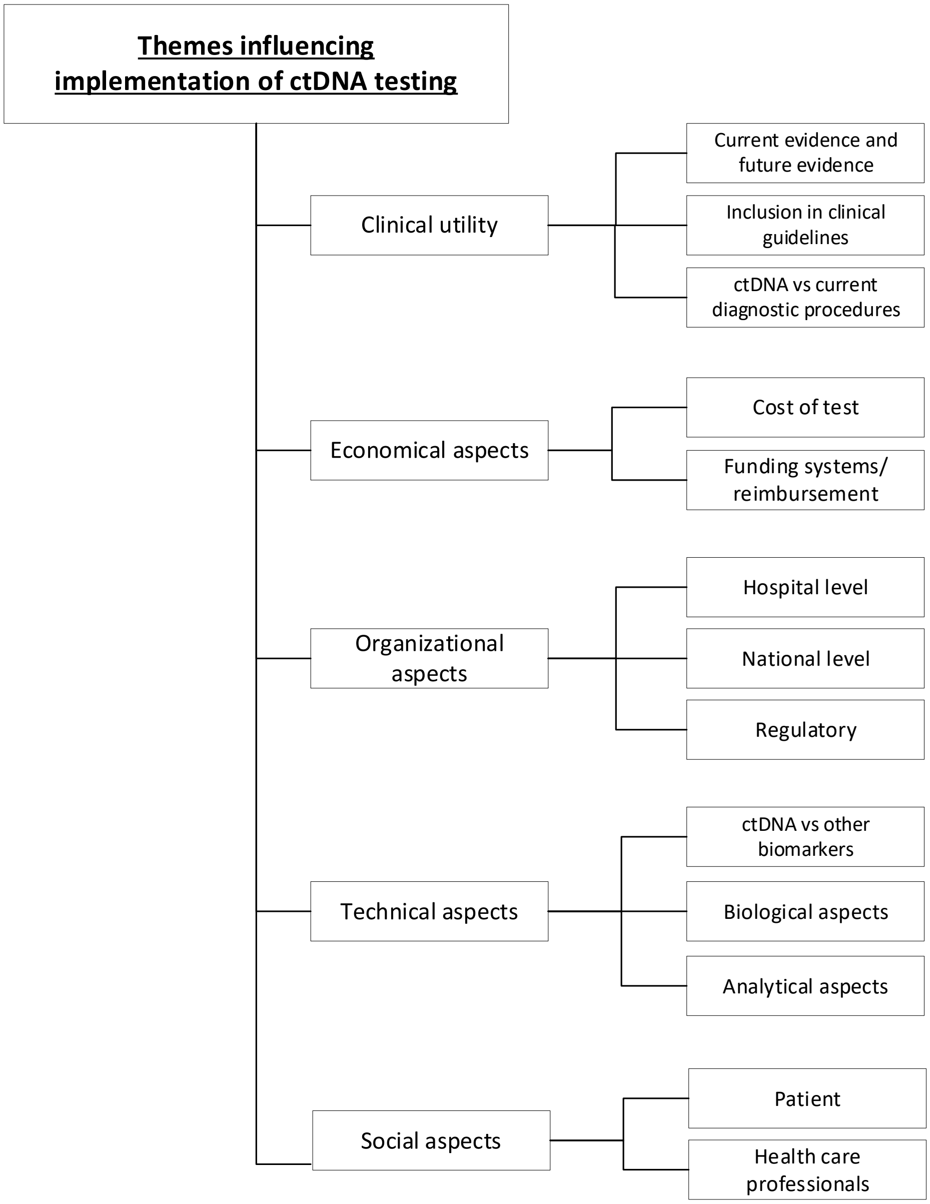

Supplementary figure 2. Identified themes (first level) and subthemes (second level) relevant for ctDNA testing implementation after categorization of the aspects derived from scoping literature review and first focus group. ctDNA, circulating tumor DNA.

Supplementary figure 3. Ranking of challenges to achieve successful implementation of ctDNA testing.

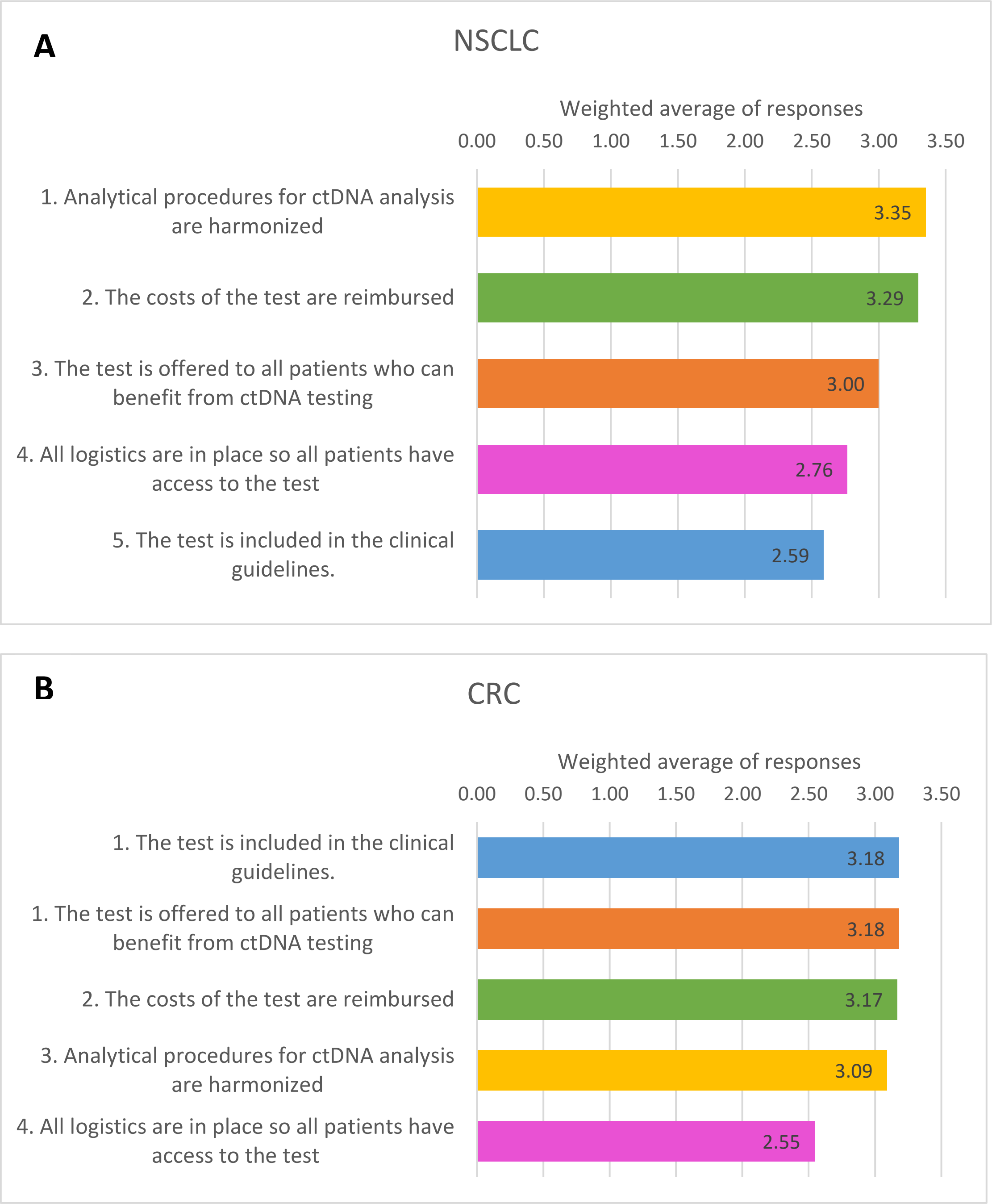

Supplementary figure 3: Answers to question number 46 in the questionnaire: Based on your knowledge and opinion, rank these 5 tracks from MOST challenging to achieve to LEAST challenging to achieve. The y axis shows the theme-specific scenarios to be ranked. X axis shows the weighted average of the responses based on the scores indicated in the methods section. **A** shows the results for NSCLC experts, **B** shows the results for CRC experts. NSCLC, Non-small cell lung cancer; CRC, colorectal cancer; ctDNA, circulating tumor DNA.

Supplementary figure 4. Positioning of ctDNA testing in the diagnostic procedures

A

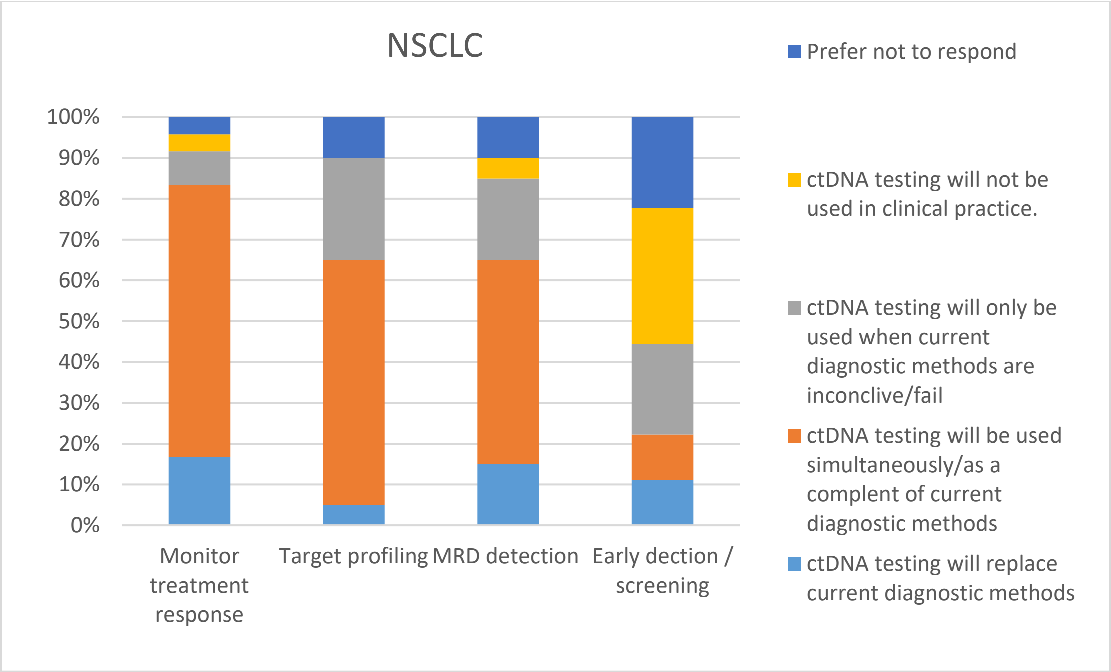

B

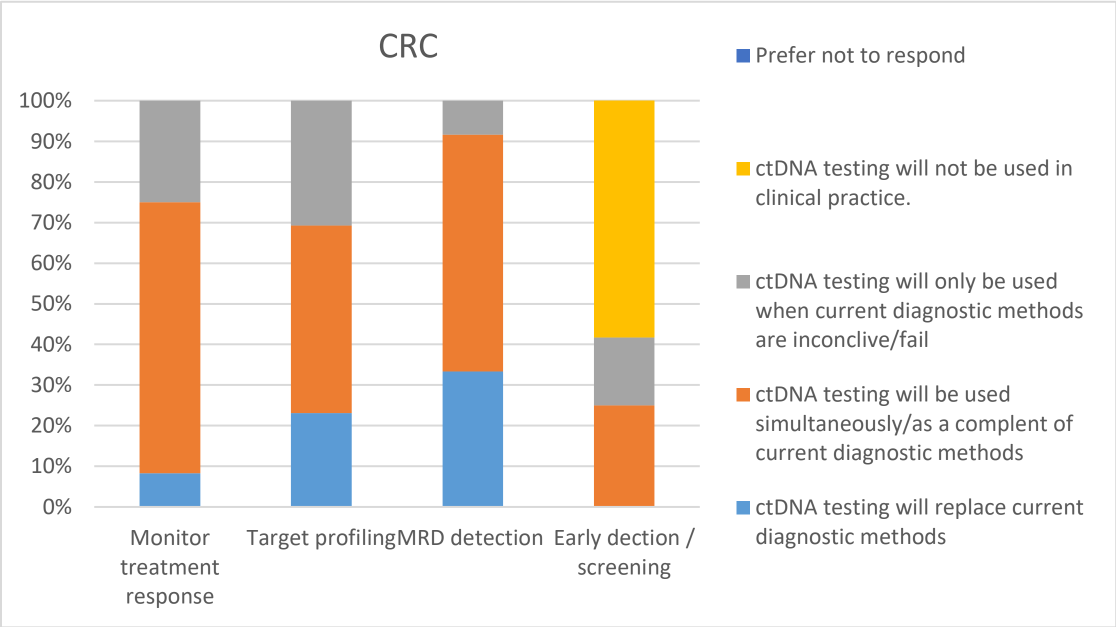

Supplementary figure 4: Answers to question number 17 in the questionnaire: "Based on your knowledge and opinion, what will be the positioning of ctDNA testing compared to current diagnostic standards within 5 years for the different applications?". X axis shows the clinical applications of ctDNA testing, y axis the percentage of respondents selecting each positioning option. **A** shows the results for NSCLC experts, **B** shows the results for CRC experts. NSCLC, Non-small cell lung cancer; CRC, colorectal cancer; ctDNA, circulating tumor DNA.

Supplementary figure 5. Role of ctDNA testing in clinical decision making.

A

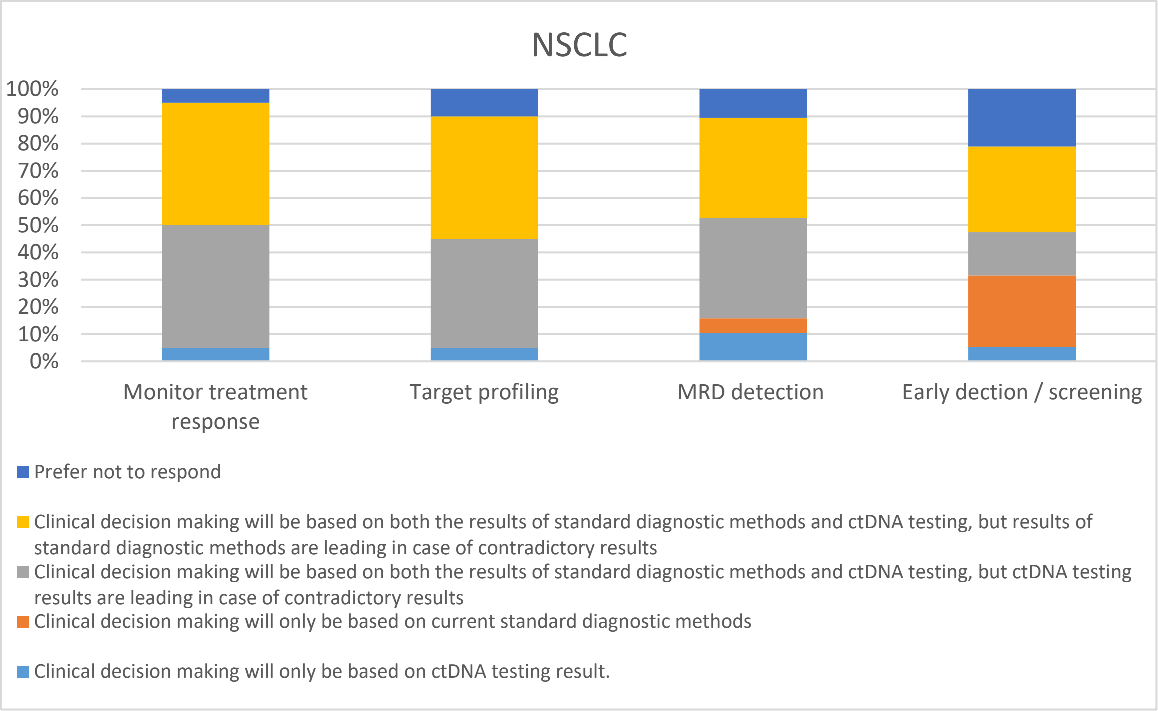

B

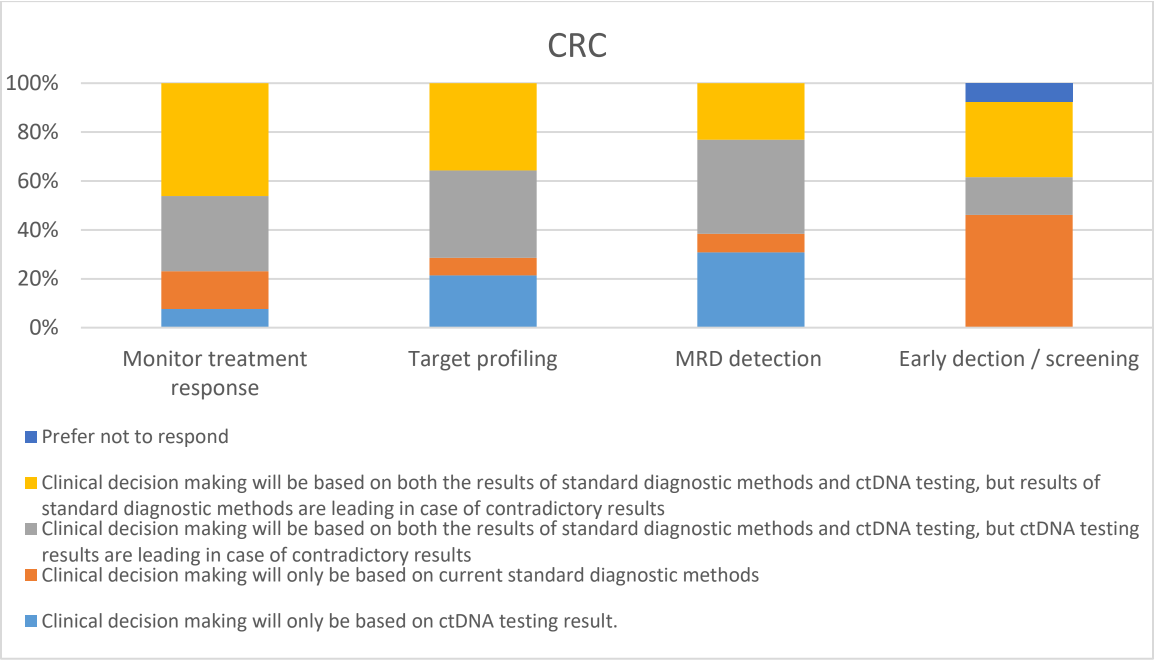

Supplementary figure 5: Answers to question number 18 in the questionnaire: "How will the results of ctDNA testing be used to inform clinical decision making within 5 years?". X axis shows the clinical applications of ctDNA testing, y axis the percentage of respondents selecting each option. **A** shows the results for NSCLC experts, **B** shows the results for CRC experts. NSCLC, Non-small cell lung cancer; CRC, colorectal cancer; ctDNA, circulating tumor DNA; MRD, minimal residual disease.

Supplementary figure 6. Expectations about cost of ctDNA testing**.**

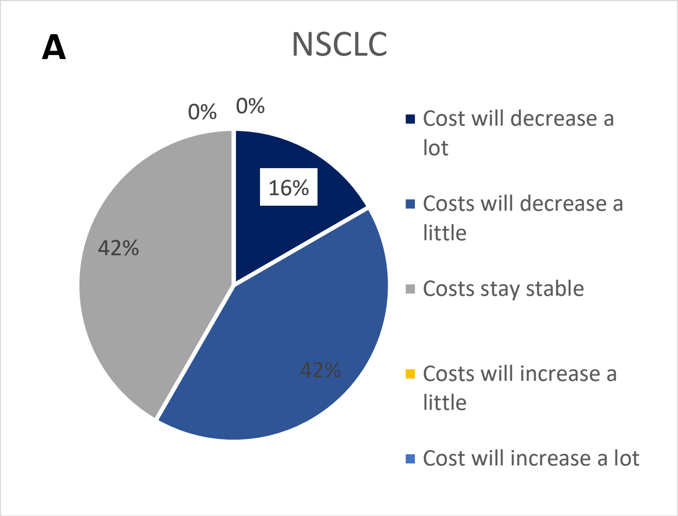

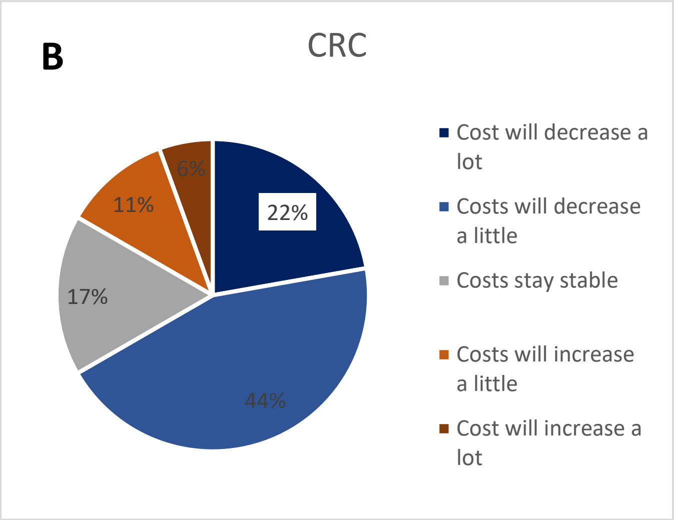

Supplementary figure 6: Answers to question number 27 in the questionnaire: "Based on your knowledge and opinion, how will the costs of ctDNA testing change within 5 years". **A** shows the results for NSCLC experts, **B** shows the results for CRC experts. All the participants answered this question. NSCLC, Non-small cell lung cancer; CRC, colorectal cancer; ctDNA, circulating tumor DNA.

Supplementary figure 7. Perception about budget restrictions for ctDNA testing.

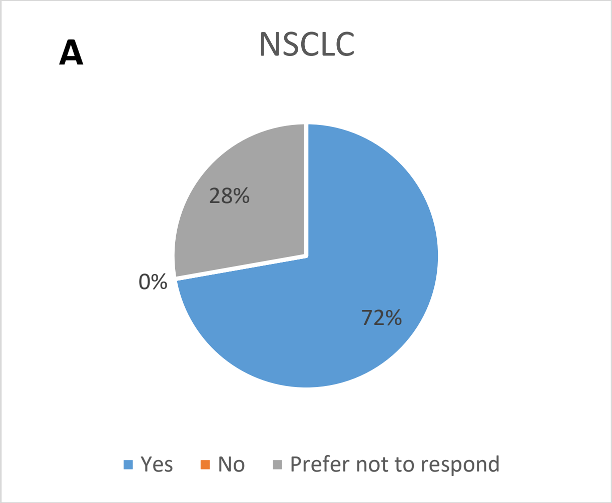

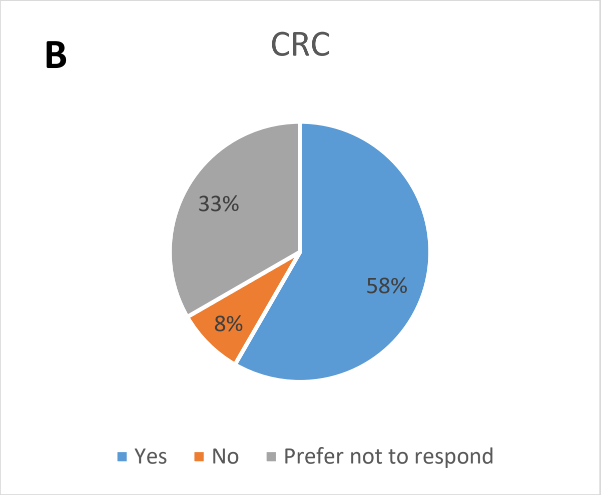

Supplementary figure 7: Answers to question number 28 in the questionnaire: "Based on your knowledge and opinion, are there currently any budget restrictions for ctDNA testing in the diagnostic setting?". **A** shows the results for NSCLC experts, **B** shows the results for CRC experts. NSCLC, Non-small cell lung cancer; CRC, colorectal cancer; ctDNA, circulating tumor DNA.

Supplementary figure 8, Opinion about centralization in one center.

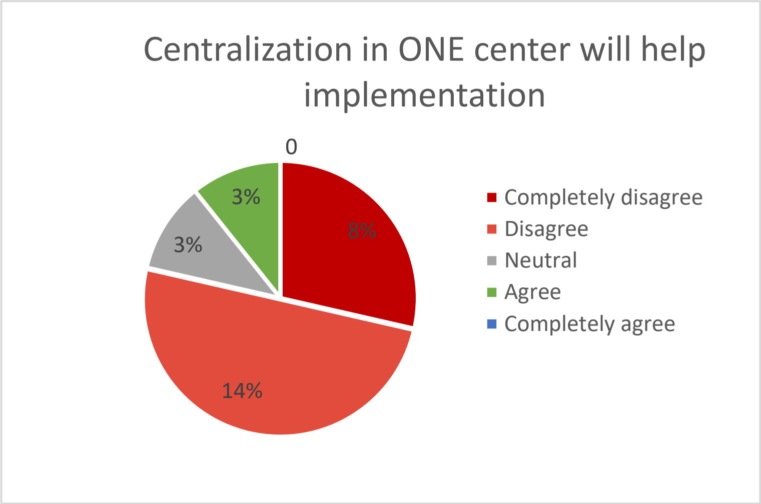

Supplementary figure 8: Answers to question number 32 in the questionnaire: "Based on your knowledge and opinion, do you agree with the following statement? It will help the implementation of ctDNA testing if only ONE center performs all ctDNA testing for all patients in the Netherlands". Results from NSCLC and CRC were pooled.

Supplementary figure 9, Opinion about patient preference for ctDNA testing over current diagnostic methods.

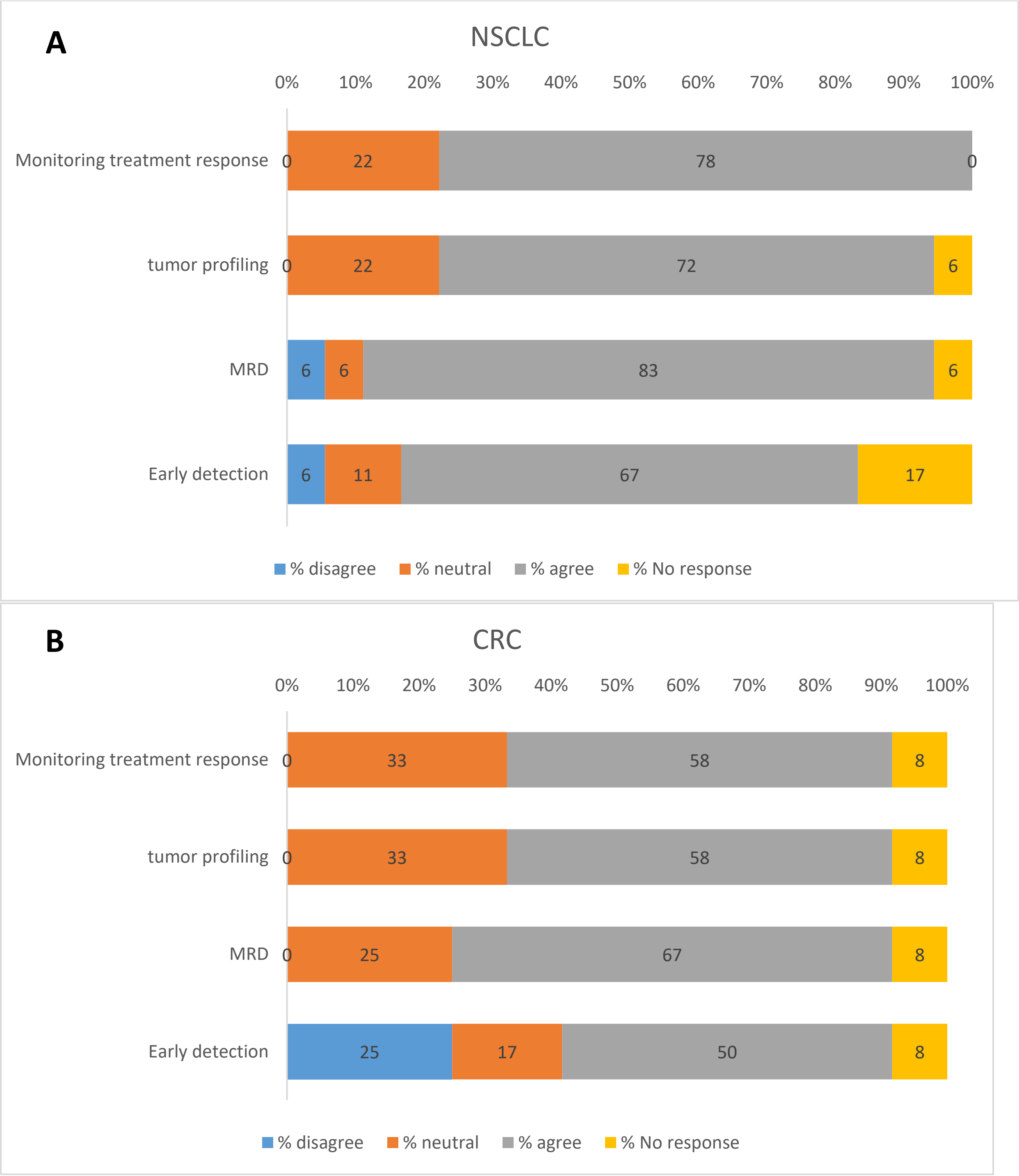

Supplementary figure 9: Answers to question number 45 in the questionnaire: "Patient will prefer ctDNA testing to standard of care diagnostics". **A** shows the results for NSCLC experts, **B** shows the results for CRC experts. NSCLC, Non-small cell lung cancer; CRC, colorectal cancer; ctDNA, circulating tumor DNA.
