# Supplementary material for: A scenario‐drafting study to explore potential future implementation pathways of circulating tumor DNA testing in oncology
Source: Mol Oncol. 2024 Jan 11;18(11):2730–42. doi: 10.1002/1878-0261.13562 (PMC11547223; doi:10.1002/1878-0261.13562)
Supplement: Supplementary file 3 — Table S1. Detailed respondent characteristics. Table S2. Detailed overview of the results for all scenarios. Table S3. Expectation about obtaining survival benefit as a result of ctDNA testing. Table S4. Aspects different to survival benefit leading to inclusion of ctDNA testing in clinical guidelines. Table S5. Ensuring patient access. Table S6. Main advantages and disadvantages of centralization. [file MOL2-18-2730-s001.pdf]

Supplementary table 1. Detailed respondent characteristics.

	NSCLC	CRC	Total
**All**	18	12	30
**Profession**			
Laboratory specialist	11	0	11
Clinical researcher	2	8	10
Fundamental researcher	1	3	4
Clinical scientist in molecular pathology	3	0	3
Policy maker	1	0	1
HTA-researcher/Health economist	0	1	1
**Place of employment**			
Academic hospital	10	7	17
Specialized cancer center	5	4	9
General hospital	2	0	2
Healthcare insurance company	1	0	1
University	0	1	1
**Years of experience with ctDNA (mean)**	5.1 years	4.4 years	4.9 years
**Level of knowledge (No knowledge/Basic knowledge/Expert knowledge) :**			
Monitoring treatment response	(0/7/11)	(1/6/5)	(1/13/16)
Tumor profiling	(1/5/12)	(0/8/4)	(1/13/16)
Minimal residual disease detection	(2/10/6)	(1/4/7)	(3/14/13)
Early detection/Screening	(3/6/9)	(3/7/2)	(6/13/11)

Supplementary table 1. NSCLC, Non-small cell lung cancer; CRC, colorectal cancer; HTA, Health Technology Assessment; ctDNA, circulating tumor DNA.

Supplementary table 2. Detailed overview of the results for all scenarios.

Scenario	Specific ctDNA application	NSCLC (n=18)		CRC (n=12)	
		Median likelihood (min. – max.)	Prefer not to respond	Median likelihood (min. – max.)	Prefer not to respond
Successful implementation					
ctDNA testing will be fully implemented	Monitoring treatment response (MTR)	82% (2-100%)	2	33.5%(0-86%)	1
	Tumor profiling (TP)	74% (13-100%)	3	50% (0-91%)	1
	MRD detection (MRD)	53% (1-99%)	3	49% (0-92%)	1
	Early detection/screening (ED/S)	25% (0-85%)	5	4.5% (1-49%)	1
Clinical utility					
ctDNA testing will be included in clinical guidelines	Monitoring treatment response (MTR)	86% (0-100%)	2	33.5% (1-100%)	1
	Tumor profiling (TP)	86% (49-100%)	3	53% (1-100%)	1
	MRD detection (MRD)	56% (0-100%)	3	44.5% (1-95%)	1
	Early detection/screening (ED/S)	30% (0-84%)	4	7% (0-46%)	1
Economical aspects					
ctDNA testing will be reimbursed	Monitoring treatment response (MTR)	79% (8-100%)	1	39% (1-89%)	1
	Tumor profiling (TP)	95% (39-100%)	2	66.5% (5-99%)	1
	MRD detection (MRD)	55% (1-100%)	3	63% (6-95%)	1
	Early detection/screening (ED/S)	20% (0-99%)	4	4.5% (0-41%)	1
The primary tumor will be sequenced as part of standard of care		81% (51-100%)	3	72% (4-100%)	1
Organizational aspects					
The logistics are in place so all clinicians can request ctDNA testing for their patients		81% (10-100%)	1	67.5% (2-99%)	1
Centralization of ctDNA testing in a few hospitals		57% (23-100%)	1	78% (49-100%)	1
Technical aspects					
The pre-analytical procedures are harmonized on a national level		68% (23-100%)	3	40% (7-99%)	2
The analytical procedures are harmonized on a national level		37% (2-79%)	2	48% (11-99%)	2
result interpretation and reporting are harmonized on a national level		76% (22-96%)	2	77% (13-93%)	1
another liquid biopsy biomarker will outperform ctDNA	Monitoring treatment response (MTR)	32% (0-93%)	3	45% (5-100%)	2
	Tumor profiling (TP)	19% (0-55%)	4	20% (1-59%)	2
	MRD detection (MRD)	28% (0-58%)	4	32% (10-50%)	2
	Early detection/screening (ED/S)	50% (0-58%)	6	50% (1-100%)	1
Social aspects					
A clinician will offer ctDNA testing to the patient, assuming it is included in the clinical guidelines		86% (31-100%)	2	85.5% (25-100%)	1
A clinician will offer ctDNA testing to the patient, assuming it is not included in the clinical guidelines		49% (5-74%)	2	19% (1-93%)	1

Supplementary table 2. NSCLC, Non-small cell lung cancer; CRC, colorectal cancer; ctDNA, circulating tumor DNA.

Supplementary table 3. Expectation about obtaining survival benefit as a result of ctDNA testing.

Survival benefit	NSCLC	CRC
Monitor treatment response	71%	27%
Target profiling	59%	45%
MRD detection	65%	82%
Early dection / screening	53%	18%
None of them	6%	0%
Prefer not to respond	6%	0%

Supplementary table 3. Answers to question 19: Based on your knowledge and opinion, in which application(s) will ctDNA testing potentially improve survival of the patients significantly? Results indicate the percentage of experts considering that ctDNA testing will potentially improve survival of the patients significantly. NSCLC, Non-small cell lung cancer; CRC, colorectal cancer.

Supplementary table 4. Aspects different to survival benefit leading to inclusion of ctDNA testing in clinical guidelines

Other clinical benefits	NSCLC	CRC
Unmet clinical need	59%	18%
High burden of tissue biopsy methods (liquid biopsies less invasive)	100%	73%
High concordance with current diagnostics	41%	45%
Improvement of Quality of life	47%	45%
Other	18%	36%

Supplementary table 4. Results to question 20: Results indicate the percentage of experts considering that that aspect could lead to inclusion of ctDNA testing in clinical guidelines. NSCLC, Non-small cell lung cancer; CRC, colorectal cancer

Supplementary table 5. Ensuring patient access.

Theme	Answer
Economical	Reimbursement
Social	Education to physicians
Social	Communication
Organizational	(Use of) existing logistics
Organizational	ctDNA testing considered standard of care/included in the guidelines
	**Total number of responses: 50**

Supplementary table 5. Answers to question 34: "Based on your knowledge and opinion, how can we ensure that every patient has access to ctDNA testing?". Answers were coded and classified in themes. Answers are presented by code, and classified in themes. In total 30 experts were exposed to this question, results from NSCLC and CRC experts are presented together.
Only answers provided by more than 10% of the respondents are included in the table. Color code: Green: >50% of the respondents report this advantage/disadvantage. Orange: >25% of the respondents report this advantage/disadvantage. Yellow: >10% report this advantage/disadvantage.

Supplementary table 6. Main advantages and disadvantages of centralization**.**

Advantages	Disadvantages
Cost reduction	Logistics
Increased expertise	Turnaround time increased
Scale efficiencies	Risk that the expertise would be limited to few centers
Quality	
Uniform analysis and reporting	
Turnaround time reduced	
Large cohorts will promote an increase of innovation and development	
**Total number of advantages mentioned: 66**	**Total number of disadvantages mentioned: 33**

Supplementary table 6. Answers to question 31: "Based on your knowledge and opinion, what would be the main advantage and disadvantage of centralizing the ctDNA testing in few hospitals?". Answers are presented by code for NSCLC and CRC together, in total 30 experts were exposed to this question. Only answers provided by more than 10% of the respondents are included in the table. Color code: Green: >50% of the respondents report this advantage/disadvantage. Orange: >25% of the respondents report this advantage/disadvantage. Yellow: >10% report this advantage/disadvantage.
